# Gut microbial dysbiosis exacerbates long-term cognitive impairments by promoting intestinal dysfunction and neuroinflammation following neonatal hypoxia-ischemia

**DOI:** 10.1080/19490976.2025.2471015

**Published:** 2025-02-26

**Authors:** Andi Chen, Chengqian Teng, Jianjie Wei, Xuyang Wu, Honghong Zhang, Pinzhong Chen, Dingliang Cai, Haitao Qian, Hui Zhu, Xiaochun Zheng, Xiaohui Chen

**Affiliations:** a Shengli Clinical Medical College of Fujian Medical University, Department of Anesthesiology, Fujian Provincial Hospital, Fuzhou University Affiliated Provincial Hospital, Fuzhou, China; b Department of Neonatal Intensive Care Unit, Fujian Provincial Hospital, Fuzhou University Affiliated Provincial Hospital, Fuzhou, China

**Keywords:** Neonatal hypoxic-ischemic brain damage, microbiota-gut-brain axis, cognitive impairments, neuroinflammation, fecal microbiota transplantation

## Abstract

Neonatal hypoxic-ischemic brain damage (HIBD) is considered as a major cause of long-term cognitive impairments in newborns. It has been demonstrated that gut microbiota is closely associated with the prognosis of various neurological disorders. However, the role of microbiota-gut-brain axis on cognitive function following neonatal HIBD remains elusive. In this experiment, the correlation analysis supported the involvement of gut microbial changes following hypoxic-ischemic (HI) insult in the development of long-term cognitive impairments. Subsequent experiment revealed the involvement of the intestinal dysfunction in the hippocampal neuroinflammation and synaptic injury. In causal relationship validation experiments, fecal microbiota transplantation (FMT) from cognitively normal rats could restore gut microbial composition, improve intestinal dysfunction, reduce the serum levels of lipopolysaccharides (LPS) and inflammatory mediators, and alleviate neuroinflammation, synaptic damage and cognitive impairments in neonatal HIBD recipient rats. Conversely, the FMT from neonatal HIBD rats could induce above adverse pathological changes in the normal recipient rats. Moreover, oral administration of anti-inflammatory agent dexamethasone (DEX) exhibited the potential to alleviate these detrimental effects in neonatal HIBD rats, with the efficacy being partly reliant on gut microbiota. Further experiment on the potential molecular mechanisms using RNA sequencing indicated a significant increase in the toll-like receptor 4 (TLR4) gene in the intestinal tissues of neonatal HIBD rats. Additionally, the interventions such as TLR4 inhibitor TLR4-IN-C34 administration, FMT, and oral DEX were demonstrated to modulate intestinal function by inhibiting the LPS/TLR4 signaling pathway, thereby exerting neuroprotective effects. Collectively, these findings underscore the contribution of gut microbial dysbiosis post HI insult in activating the LPS/TLR4 signaling pathway, triggering intestinal inflammation and dysfunction, exacerbating systemic inflammation, and consequently worsening synaptic and cognitive impairments in neonatal HIBD rats. Hence, rectifying gut microbial dysbiosis or regulating intestinal function may represent a promising strategy for alleviating long-term cognitive impairments in neonates affected by HIBD.

## GRAPHICAL ABSTRACT

**Figure:**
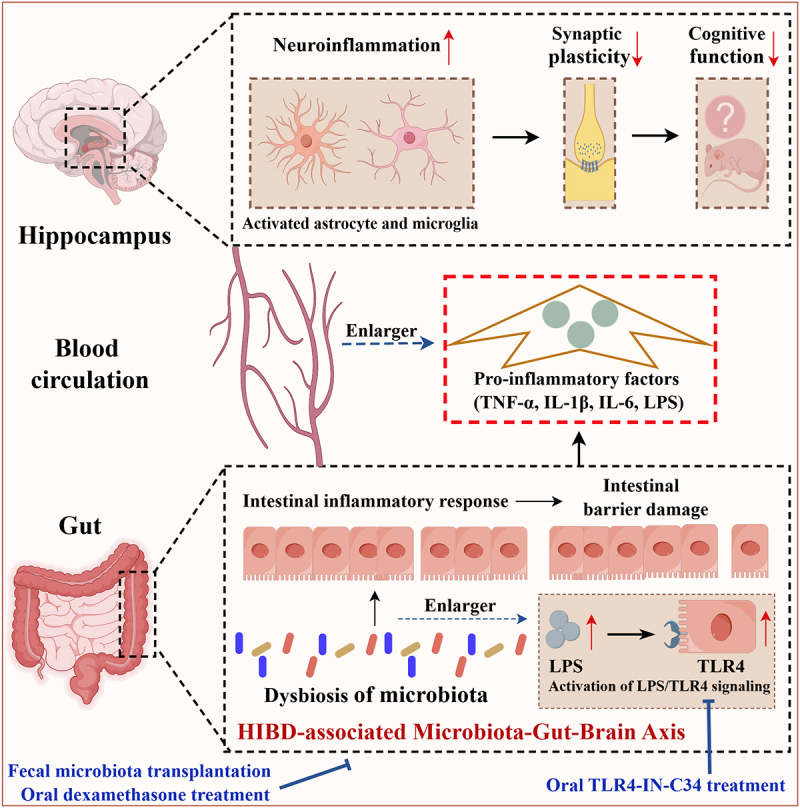

## Introduction

Neonatal hypoxic-ischemic brain damage (HIBD) represents a predominant cause of neurocognitive disorders in children, profoundly impacting their physical and mental development and quality of life, while imposing significant burdens on families and society.^1,2^ Brain magnetic resonance imaging (MRI) of human neonates with HIBD^3^ and HIBD animal models^4^ has revealed that among the various brain regions affected by hypoxic-ischemic (HI) insult, the hippocampal damage is particularly severe. This condition frequently stems from the intense and sustained neuroinflammation in the hippocampus following HIBD, culminating in significant synaptic impairments and neuronal damage, which represents one of primary pathological mechanism underlying the long-term cognitive impairments attributed to HIBD.^5–7^ Nevertheless, the upstream factors responsible for triggering neuroinflammation in the hippocampus following HIBD remain incompletely understood, and effective preventive measures are still to be discovered.

Increasing evidence^8–10^ suggests that the gut microbiota can influence brain function and behavior through the microbiome-gut-brain axis, offering a novel perspective for researching a range of diseases associated with brain function. The research^11^ on stroke indicates that gut microbiota can influence the neurological functional outcome in mice after cerebral ischemia. Additionally, available works demonstrate that fecal microbiota transplantation (FMT) from healthy individuals can mitigate the activation of microglia, reduce neuroinflammation, and improve behavior across various neurological disorders, such as Parkinson’s disease (PD),^12^ Alzheimer’s disease (AD)^13^ and perioperative neurocognitive disorders (PND).^10^ Especially, a recent work by Drobyshevsky et al.^14^ on neonatal HIBD animal model investigated the manipulation of gut microbial composition using an established murine model of perinatal HI insult. Their findings suggest that gut microbiota may influence neuroinflammatory responses and brain injury following neonatal HI insult, hinting at a potential role of gut microbiota in determining the neurocognitive outcomes of HIBD in humans. However, it remains unclear how gut microbial dysbiosis mediates neuroinflammation, potentially exacerbating synaptic damage and long-term cognitive impairments following HIBD.

The gut microbiota can directly influence intestinal epithelial cells, interfering their gene expression and subsequently changing intestinal function and related signaling pathways.^15^ Furthermore, recent studies^10,16,17^ have indicated that intestinal dysfunction induced by gut microbial dysbiosis, including intestinal inflammation and intestinal barrier impairments, is closely associated with outcomes of the various neurological diseases, such as PND and AD. Additionally, excessive intestinal inflammatory responses can further exacerbate the intestinal barrier damage, known as “leaky gut,” allowing more harmful substances, such as gut microbiota-derived Lipopolysaccharide (LPS) and intestinal epithelial cells-secreted inflammatory cytokines (tumor necrosis factor-α, TNF-α; interleukin-6, IL-6; interleukin-1β, IL-1β), to enter the bloodstream, thereby promoting neuroinflammation and impairing neural functions. However, it remains unclear if gut microbial dysbiosis after HIBD may cause intestinal dysfunction, increase pro-inflammatory mediators in the blood, and induce hippocampal neuroinflammation, as well as the underlying molecular mechanisms.

In this study, we investigated the correlation between gut microbial dysbiosis following HI insult and HIBD-induced long-term cognitive impairments, and established the role of microbiota-gut-brain axis in regulating intestinal function and neuroinflammation after HIBD. Specifically, employing neonatal rats model of HIBD, we observed a significant correlation between changes in gut microbiota composition and severity of cognitive impairments after HI insult. Subsequently, we examined the intestinal inflammatory responses, intestinal barrier integrity, levels of pro-inflammatory mediators in peripheral circulation, hippocampal neuroinflammation, synaptic injury and neural damage following HI insult to explore the potential mechanisms of the microbiota-gut-brain axis in neonatal HIBD. Correlation analysis revealed significant links between intestinal dysfunction and both hippocampal neuroinflammation and synaptic injury. In the causal relationship validation experiments, the FMT from cognitively normal rats was found to restore gut microbial composition, improve intestinal function, reduce serum levels of LPS and inflammatory mediators, and alleviate neuroinflammation, synaptic damage, and cognitive impairments in the neonatal HIBD recipient rats. Conversely, the FMT from neonatal HIBD rats induced similar adverse pathological changes in the normal recipient rats. In addition, we demonstrated that oral anti-inflammatory agent dexamethasone (DEX) treatment could exert neuroprotective effects by partially reducing intestinal inflammation and improving gut microbial dysbiosis. We further revealed that the intestinal LPS/toll-like receptor 4 (TLR4) signaling pathway was a potential molecular mechanism of the microbiota-gut-brain axis following HI insult, regulating intestinal function and systemic inflammation. Collectively, our study provides the new insights for developing potential therapeutic strategies for long-term cognitive impairments induced by the neonatal HIBD from the microbiota-gut-brain axis perspective.

## Materials and methods

### Animals and experimental design

The handling of laboratory animals adhered strictly to the Guide for the Care and Use of Laboratory Animals. Our laboratory acquired pregnant Sprague-Dawley rats, specific pathogen-free (SPF) strain, at a gestational age of 13 days from Fujian Medical University (Fuzhou, China). Appropriate transport containers were selected to maintain optimal temperature and humidity within the transport vehicle, while efforts were made to reduce noise and vibrations during transit, ensuring a transport duration of under 1 hour. Upon their arrival at our facility, the pregnant rats were placed in a calm single-cage environment conducive to acclimatization, where temperature conditions were regulated, and a constant supply of food and water was provided while minimizing potential stressors. Daily monitoring of the pregnant rats’ delivery status was conducted to accurately determine gestational age and postnatal pup age. Throughout the experiment, neonatal rats were housed in cages with their littermates and nursing mothers under regulated temperature and lighting conditions (12 hours of light/12 hours of darkness), ensuring unrestricted access to food and water. Neonatal rats of both sexes from each litter were used in this study. To mitigate sex-related bias, an equal distribution of female and male pups was maintained within each experimental group. The Animal Care and Use Committee at Fujian Provincial Hospital (Fuzhou, China) granted ethical approval for this research (Approval No: IACUC-FPH-SL-20230825[0088]).

**Figure 1.**
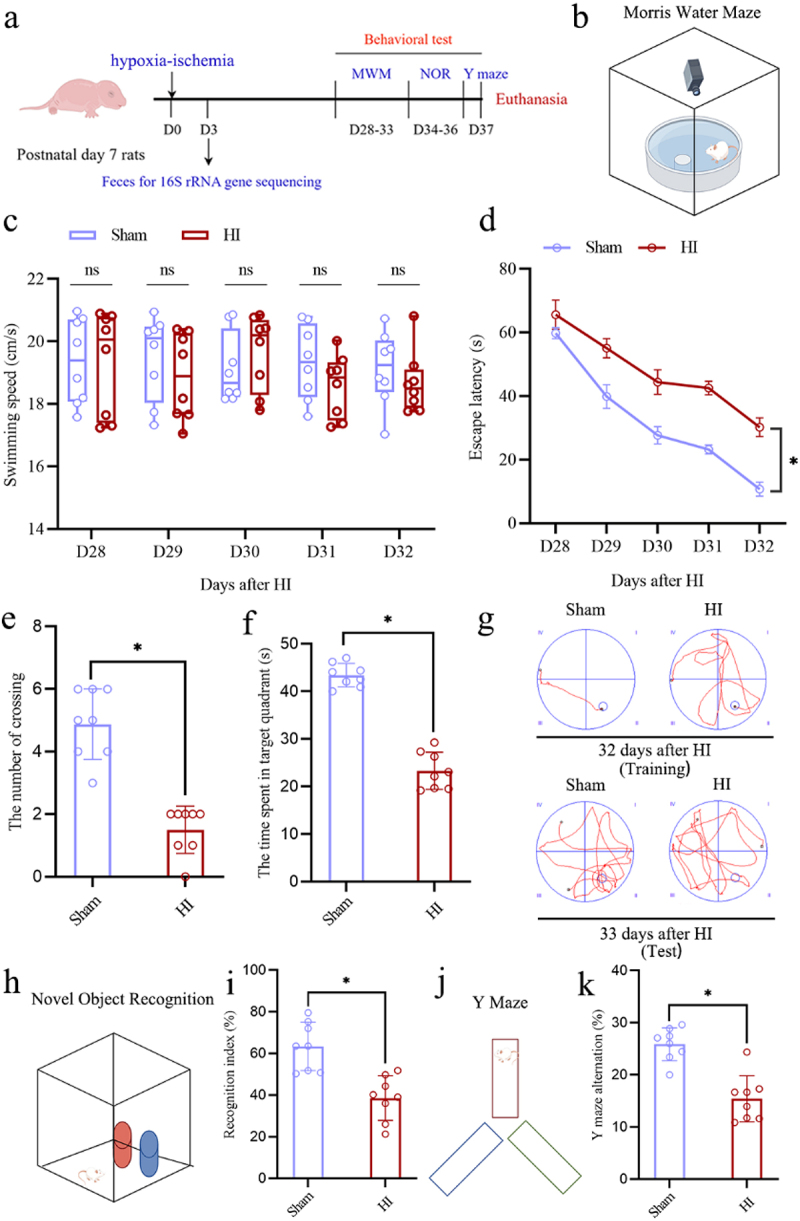
HI insult induced severe long-term cognitive impairments in the neonatal rats. a The experimental flow chart. b Schematic diagram of the MWM test. c, d the swimming speed and the escape latency of rats in the MWM test from 28 to 32 days after HI insult. e, f The number of crossing the platform times and the time spent in the target quadrant of rats in the MWM test on the 33 days after HI insult. g Representative swim tracks of rats from the place navigation trial on the 32 days after HI insult and the probe test on the 33 days after HI insult. h Schematic diagram of the nor test. i The recognition index in nor test of rats on the 36 days after HI insult. j Schematic diagram of the Y maze test. k The rate of Y maze alternation of rats on the 37 days after HI insult. HI, hypoxic-ischemic; MWM, Morris water maze; NOR, novel object recognition; *n* = 8, per group; *p < 0.05, ns means no significant.

In the initial experiment (Figure 1(a)), postnatal day 7 rats were chosen and randomly sorted into two groups: (1) the sham group, where rats underwent anesthesia and their left carotid artery was exposed but not ligated or subjected to subsequent hypoxic intervention, and (2) the HI insult group, where the Rice-Vannucci modeling method was utilized to create an animal model of HIBD. On the 3rd day after the HI insult, fecal samples were collected from both groups of neonatal rats for 16S rRNA sequencing, aiming to investigate any alterations in gut microbial composition during the acute phase after HI insult. Cognitive behavioral assessments, including the Morris water maze (MWM), novel object recognition (NOR), and Y-maze test, were performed 28–37 days post-HI insult. To explore the potential microbiota-gut-brain axis mechanisms underlying long-term cognitive impairments after HI insult, the colonic tissues, serum, and hippocampal tissues were collected on the 3rd day post-HI insult (Figure 4(a)). Furthermore, various techniques including Hematoxylin and Eosin (HE) staining, immunofluorescence (IF) staining, immunohistochemistry (IHC) staining, enzyme-linked immunosorbent assay (ELISA), transmission electron microscopy (TEM), Golgi staining, and Nissl staining were utilized to evaluate the integrity of the intestinal barrier, the inflammatory response in the intestine, serum levels of pro-inflammatory mediators, activation of neuroinflammatory glial cells in the hippocampus, synaptic structure, and the quantity and morphology of hippocampal neurons following HIBD. Additionally, the correlation analysis was conducted to determine the possible link between gut microbial dysbiosis and long-term cognitive impairments post-HI insult, and between intestinal dysfunction and hippocampal pathological changes.

To further validate the potential causal relationship between gut microbial dysbiosis following HI insult and long-term cognitive impairments, various FMT strategies, including transferring fecal microbiota from rats in the sham group to recipient rats in the HI group and vice versa (Figure 7a), were conducted. Transplantations were performed daily from the 7th postnatal day to the 3rd day post-HI insult. The effectiveness of FMT was validated through quantitative PCR (qPCR) analysis, followed by evaluations of intestinal inflammation, integrity of the intestinal barrier, serum levels of pro-inflammatory mediators, activation of neuroinflammatory glial cells in the hippocampus, synaptic structure, and long-term cognitive function.

Increased intestinal inflammation is a primary factor of barrier impairments.^10^ DEX, a conventional anti-inflammatory drug, can significantly mitigate the intestinal barrier damage caused by diverse etiologies through exerting a protective anti-inflammatory effect.^10,18,19^ Moreover, available literatures suggest that the gut microbiota is a key regulatory factor for the observed anti-inflammatory effects on intestinal tissues after DEX treatment.^20,21^ Thus, the observed beneficial effects of FMT on cognitive impairments induced by HI insult encouraged us to determine the potential impacts of oral DEX treatment within a similar framework. Previous research^10^ indicated that an oral dose of DEX 1 mg/kg was safe in the mouse models of other neurological diseases and beneficial for gut microbiota, intestinal function, and cognitive function. In our pilot experiments (see Supplementary note 1 and Supplementary Figure S1), we investigated oral DEX dosage gradients in neonatal HIBD rats and demonstrated that an oral dose of DEX 1 mg/kg was safe and effective. Then, postnatal day 7 rats were randomly assigned to the sham group, HI group and HI+DEX group (Figure 9(a)). The rats in the HI+DEX group were administered DEX (ST1254, beyotime, China) orally at a dosage of 1 mg/kg daily from the 7th postnatal day to the 3rd day after HI insult. Subsequently, intestinal inflammation, intestinal barrier integrity, serum levels of pro-inflammatory mediators, activation of hippocampal neuroinflammatory glial cells, synaptic structure, and long-term cognitive function were tested. To further verify whether the gut microbiota were also the key mediators for the neuroprotective effect of oral DEX, the FMT was conducted for validation. Fecal microbiota from both the HI group and HI+DEX group donor rats were transplanted into the HI group recipients daily from the 7th day after birth to the 3rd day post HI insult (Figure 10(a)). The efficacy of FMT was confirmed by the qPCR, followed by assessments of activation of hippocampal neuroinflammatory glial cells, synaptic structure, and long-term cognitive function.

To further explore the potential molecular mechanisms of intestinal inflammation following HIBD, a high-throughput RNA-Seq was conducted to determine the related genes and molecular pathways (Figure 11(a)). Based on the literature reports,^22–24^ we selected TLR4-IN-C34, an orally active and specific TLR4 inhibitor, for further study. Furthermore, in our pilot experiments (see Supplementary note s2 and Supplementary figure s2), we investigated the dose-response relation of oral TLR4-IN-C34 in the neonatal HIBD rats and demonstrated that an oral dose of TLR4-IN-C34 1 mg/kg was safe and effective. Then, the rats in the HI+TLR4-IN-C34 group were administered TLR4-IN-C34 (HY-107575, MedChemExpress, USA) orally at a dosage of 1 mg/kg daily from the 7th postnatal day to the 3rd day after HI insult. Subsequently, intestinal TLR4 expression, intestinal function, systemic inflammation, synaptic structure, and long-term cognitive function were determined. Furthermore, additional experiments were performed to determine whether the neuroprotective effects of FMT and oral DEX interventions were related to the regulation of intestinal LPS/TLR4 signaling pathway.

To simulate the clinical scenario of oral drug administration, the oral DEX and TLR4-IN-C34 method used in this study followed the conventional approach, that is, the drug powders was added to the rats’ regular sterile drinking water.^25,26^ Considering that 7–10-day-old rats are not yet able to drink water independently, we added DEX and TLR4-IN-C34 powders into centrifuge tubes with 2 ml of sterile drinking water, thoroughly mixed them through shaking, and slowly administered them orally using a syringe while monitoring swallowing to ensure accurate dosing. To ensure experimental consistency, neonatal rats in the other groups received equivalent volume of sterile drinking water.

For histological analyses, five tissue sections from different levels of consecutive slices from the brain and intestine tissues of each animal were selected and evaluated at three positions in each section relevant to the experimental objectives. For each sample, a minimum of ten measurements were taken from non-overlapping, well-oriented areas. The average counts from these evaluations were then included in the final statistical analysis. Five animals from each group were included in the analysis. The number of animal samples used in the pilot study and in different experimental groups at various stages of the main study are shown in Supplementary Tables S1 and S2.

### Neonatal HIBD animal model

In this study, the Rice – Vannucci model approach was performed to construct a neonatal HIBD animal model, following the protocols outlined in our previous research.^27^ Initially, on postnatal day 7, the left common carotid artery of SD rats was ligated under anesthesia with 3% isoflurane. Following a recovery period of 1 hour in the proximity of their dams, the neonatal rats were then placed in a hypoxic chamber with 8% O_2_ at a temperature of 37°C for 2 hours. This procedure resulted in HI insult in the neonatal rats.

### Cognitive behavioral tests

Cognitive behavioral tests, including the MWM, NOR, and Y-maze tests, were conducted in accordance with previous reports.^1,10,28–30^

### MWM

The MWM test was performed from the 28th to the 33rd day post HI insult to evaluate the cognitive functions of rats. The MWM test spanned 6 days, starting with a place navigation test for the initial 5 days, followed by a probe trial on the final day. All tests were completed consistently between 9:00 a.m. and 3:00 p.m. to mitigate disruptions from diurnal variations and light influences on experimental results. A video camera positioned above the pool, segmented into four quadrants with a submerged platform (12 cm in diameter) in one quadrant, recorded rat movements. Prior to the formal tests, rats were allowed 90 seconds of free swim in the pool. During the place navigation phase, rats, starting against the pool wall, were given 90 seconds to locate the escape platform. The rats unable to find the platform within this timeframe were guided to it and remained there for 30 seconds. Four trials were conducted daily for five days, and the average escape latency was recorded to evaluate spatial learning and memory capabilities. On the 6th day, during the probe trial, the hidden platform was removed, and rats were allowed to swim for 90 seconds; spatial memory was assessed by observing the number of platform crossings and time spent in the target quadrant.

### NOR

The NOR test was performed from the 34th and 36th days post HI insult to assess the cognitive functions of rats. Beginning on the 34th day post HI insult, all rats designated for the NOR test were placed in a box-shaped space for a 10-minute acclimation period. The following day, a log-colored cylindrical wooden block (Object A) and a blue cylindrical wooden block (Object B) were positioned at opposite ends of the same wall within the box-shaped space. Rats were then introduced into the box-shaped space with their backs to these objects to commence a training session aimed at exploring Objects A and B, which was documented via an overhead video recorder for 10 minutes. On the 36th day post HI insult, we substituted Object B with a red triangular wooden block (Object C) and recorded the rats’ exploratory behavior toward this new object for an additional 10 minutes. Following video review, the duration of time that the rats spent in the novel object (Object C) was noted. The Recognition Index (RI) was subsequently calculated as follows: RI = [(time spent exploring the novel object)/(total time spent exploring both objects)] × 100%.

### Y maze

The Y maze test was performed on the 37th days post HI insult to assess the cognitive functions of rats. The maze consisted of three arms. During the test, each test rat was placed in one arm and permitted to move freely among the arms for a duration of five minutes. The alternation rate was calculated using the formula: actual alternations/maximum possible alternations × 100%. The term “actual alternations” refers to rats entering the three arms consecutively and is recorded as one alternation, while “maximum possible alternations” is defined as the total number of arm entries minus 2.

### 16S rRNA sequencing and bioinformatic analysis

Fresh fecal samples from rats of each group were collected on the 3rd day post HI insult. Bacterial genomic DNA was then extracted from these samples using the QIAamp Power Fecal Pro DNA Kit (QIAGEN, Germany), adhering to the manufacturer’s instructions. Subsequently, the V3-V4 regions of the microbial 16S rRNA genes were amplified using specific primers (forward: 5′-CCTACGGGNGGCWGCAG-3′; reverse: 5′-GGACTACHVGGGTATCTAAT-3′). Amplification conditions included an initial step at 95°C for 5 minutes, followed by 30 cycles of denaturation at 72°C for 1 minute, and a final extension at 72°C for 7 minutes. Equal concentrations of the quantified amplicons were pooled for Illumina MiSeq sequencing (Illumina, Inc., CA, USA). Gene Denovo (Guangzhou, China) conducted DNA extraction, quality assessment, library construction, and high-throughput sequencing, and further collaborated with us to process and analyze the 16S rRNA sequencing data. In brief, FASTP (V0.18) was employed to filter the clean reads, specifically excluding those with an average Phred score below 20, those containing adapter sequences, and those with more than 10% unknown nucleotides (N). Subsequently, Operational Taxonomic Units (OTUs) were clustered at a similarity threshold exceeding 97% utilizing UPARSE (V9.2.64). The OTU sequences thus obtained were annotated to classify taxa using the SILVA database (V138.1).^31–33^ Then using the OmicShare tools (https://www.omicshare.com/tools/) provided by Gene Denovo to analyze α-diversity, β-diversity, analysis of similarity (ANOSIM), composition of gut microbiota, linear discriminant analysis effect size (LEfSe), and indicator species analysis.

### HE staining

The colonic tissues from each group were collected on the 3rd day post HI insult. The rats were anesthetized and subjected to cardiac perfusion with saline, followed by fixation with paraformaldehyde (PFA). The colons were then excised, sectioned into 1 cm lengths, fixed in 4% PFA for 24 hours, and embedded in paraffin. Following embedding, sections measuring 4-μm in thickness were cut, deparaffinized, and stained with hematoxylin and eosin using standard protocols. The severity of colon injury was assessed employing a modified Chiu’s scoring system, which focuses on changes in the villi and glands of the intestinal mucosa.^34^

### IF and IHC staining

Brain and colon tissues from each group were collected on the 3rd day post HI insult. Rats were anesthetized and underwent cardiac perfusion with saline, followed by fixation with PFA. Subsequently, the tissues were removed, fixed again, and dehydrated in a 30% sucrose solution. Both the IF and IHC staining analyses were performed as described in previous studies.^1,35^ For IF staining of the colon, Occludin (1:200, ab216327, Abcam, USA) and ZO-1 (1:200, ab221547, Abcam, USA) antibodies were used to detect the intestinal barrier integrity,^10^ and TLR4 (1:50, sc -293,072, Santa Cruz, USA) antibodies was used to detect the intestinal TLR4 expression. According to literature reports,^36,37^ IL-17a and IL-22 are initiators of inflammatory responses in intestinal tissue, which can trigger a series of inflammatory cascades. Hence, for IHC staining of the colon, IL-17a (1:200, ab214588, Abcam, USA) and IL-22 (1:200, ab203211, Abcam, USA) antibodies were used to detect the inflammatory response of intestine. Brain sections were stained for IF using IBA-1 (1:100, EPR16589, Abcam, USA) and GFAP (1:200, ab279289, Abcam, USA) to detect hippocampal neuroinflammatory response. All stained sections were randomly assigned for independent analysis by observer blind to the experimental conditions. Image-Pro Plus 6.0 software facilitated the analysis and export of IF and IHC measurement data.

### ELISA

Serum and fecal samples from each group were collected on the 3rd day following the HI insult. Pre-coated plates equipped with specific capture antibodies for LPS (Jianglai Industrial Limited, China), TNF-α (E-EL-R2856, Elabscience, China), IL-6 (E-EL-R0015, Elabscience, China), and IL-1β (E-EL-R0012, Elabscience, China) were prepared. To determine the fecal and serum LPS levels and the serum levels of inflammatory mediators including TNF-α, IL-6, and IL-1β, the respective standards and target samples were placed into corresponding wells. The plates were incubated for 24 hours at room temperature, facilitating the binding of the target proteins to the capture antibodies. Subsequently, a substrate solution for horseradish peroxidase (HRP) was added to each well, with the color development meticulously monitored over a 10 to 30 minutes interval. The enzymatic reaction was terminated by the addition of a stop solution, which changed the solution’s color to yellow. Absorbance measurements were obtained using a microplate reader.

### TEM

Brain tissues from each group were obtained on the 3rd day following the HI insult. These tissues were then fixed with 2.5% glutaraldehyde for 1 hour at room temperature and subsequently dehydrated in ethanol for 10 minutes. Following dehydration, the tissues were embedded in epoxy resin, sectioned, and stained with uranyl acetate and lead citrate. The hippocampal microstructure was examined using TEM (JEM-2100), which allowed for the observation of ultrastructural morphological changes in hippocampal synapses.

### Golgi staining

Brain tissues from each group were collected on the 3rd day following the HI insult for Golgi-Cox staining. The dissected brains were submerged in Golgi-Cox solution containing 5% potassium dichromate, 5% mercuric chloride, and 5% potassium chromate for 35 days. Following immersion, the brains were placed in a 20% sucrose solution overnight and then sectioned coronally at 150 μm using a freezing microtome. The staining process included two washes with distilled water, a 2-minute treatment with 5% sodium thiosulfate, and another two washes with distilled water. The sections were dehydrated through sequential immersion in 70%, 90%, and 100% ethanol, followed by xylene, and then rinsed three times with bidistilled water. Images of the stained sections were acquired using a Nikon E600 camera.

### Nissl staining

Brain tissues from each group were harvested on the 3rd day post HI insult for Nissl staining, a technique applied to assess neuronal loss and neuron morphology in the hippocampus’s CA1 region. Following paraffin embedding and sectioning, the tissue sections underwent dewaxing and dehydration before being stained with Cresyl violet acetate for 5 to 10 minutes. This was succeeded by two quick, 10-second rinses in distilled water. The sections were then subjected to sequential dehydration through ethanol and xylene for 5 minutes each before being mounted on glass slides. The morphology, quantity, and thickness of the neurons in the hippocampus’s CA1 region were observed and analyzed using light microscopy.

### FMT

This study implemented three FMT strategies. The first strategy involved transferring fecal microbiota from sham group and HI group donor rats to sham group recipient rats. The second strategy involved transferring fecal microbiota from HI group and sham group donor rats to HI group recipient rats. The aim of the above two FMT strategies was to verify the causal relationship between gut microbiota and long-term cognitive impairments in neonatal HIBD. The third strategy involved transplanting fecal microbiota from HI group and HI+DEX group donor rats to HI group recipient rats to observe whether gut microbiota is a key mediator for the neuroprotective effect of DEX in neonatal HIBD rats. Following the methodology outlined by Deshmukh et al.^38^ pregnant rats received sterile drinking water containing a combination of five antibiotics (ampicillin, gentamicin, metronidazole, vancomycin, neomycin) from the 15th day of gestation to the 5th day post-birth to generate recipient rats with depleted gut microbiota, facilitating successful colonization by the transplanted microbiota. Donor and recipient rats for each FMT strategy were synchronized and housed separately in sterile environments. Fecal samples from donor rats were collected daily from postnatal day 7 until the 3rd day post-HI insult and transplanted to age-matched recipient rats. In line with the methodology outlined by He et al.^39^ fresh fecal pellets were collected immediately after defecation, diluted in 100 mg/ml sterile saline, stored correctly, and administered to the recipients via gavage in a sterile environment.

### *Quantification of* f_Enterobacteriaceae *and* f_Akkermansiaceae *of feces*

Two representative bacteria with significant differences in the gut microbiota between the sham and HI groups were identified from the 16S rRNA data, that is, *f_Enterobacteriaceae* and *f_Akkermansiaceae*. Therefore, after FMT, fresh feces were collected from the rats and quantification of *f_Enterobacteriaceae* and *f_Akkermansiaceae* was performed using the qPCR to assess the effect of the FMT. Total genomic DNA was extracted from fecal samples using the Stool DNA Kit (TransGen Biotech Co, China). The DNA templates were diluted to a concentration of 12.5 ng/µL using RNase-Free Distilled Water. Subsequently, 2 µL of this dilution, corresponding to 25 ng of total DNA, was used in triplicate for analysis. For *f_Enterobacteriaceae*^40^ identification, the forward primer 5′-CATTGACGTTACCCGCAGAAGAAGC-3′ and the reverse primer 5′-CTCTACGAGACTCAAGCTTGC-3′ were used. Similarly, for *f_Akkermansiaceae*^41^ detection, the forward primer was 5′-CAGCACGTGAAGGTGGGGAC-3′ and the reverse primer was 5′-CCTTGCGGTTGGCTTCAGAT-3′. Both sets of primers were utilized in conjunction with SYBR Green Nucleic Acid Gel Stains (ABclonal, China), adhering to the manufacturer’s protocol. qPCR analyses were conducted on the ABI QuantStudio 3 Real-Time PCR System (Applied Biosystems, Carlsbad, CA, USA). The reaction mixtures, with a total volume of 20 µL, underwent an initial denaturation at 95°C for 3 minutes, followed by 40 cycles of denaturation at 95°C for 5 seconds and annealing/extension at 60°C for 30 seconds. A melting curve analysis was performed to ensure the specificity of the amplification. To quantify absolute 16S rRNA gene copy numbers in the samples, standard curves were generated from serial dilutions of plasmid DNA encompassing a conserved sequence of *Enterobacteriaceae* and *Akkermansiaceae*.

### High-throughput RNA-Seq of intestinal tissues

Colonic tissues from each group were collected on the 3rd day post HI insult. Total RNA was extracted utilizing the TRIzol reagent kit (Invitrogen). The integrity and quality of the RNA were confirmed by RNase-free agarose gel electrophoresis via the Agilent 2100 Bioanalyzer (Agilent Technologies). Enriched mRNA was then reverse-transcribed into cDNA with the help of DNA polymerase I, RNase H, dNTPs, and appropriate buffer. The resulting cDNA fragments were purified and underwent end-repair using the QiaQuick PCR extraction kit (Qiagen). Following the addition of poly(A) tails, Illumina sequencing adapters were attached to the fragments. Agarose gel electrophoresis was subsequently used to select the PCR amplification products, which were then sequenced using the NovaSeq6000 by Gene Denovo Biotechnology Co.

### Western blot analysis

Colon tissues from each group were collected on the 3rd day post HI insult. The tissues were homogenized in a lysis buffer containing protease inhibitors. Proteins (40 μg/well) were separated by the SDS-PAGE and then transferred onto polyvinylidene fluoride (PVDF) membranes. The membranes were incubated overnight at 4°C with primary antibodies: anti-TLR4 (1:500, sc -293,072, Santa Cruz, USA). At the next day, the membranes were incubated with secondary antibodies for 2 hours at room temperature. Protein bands were detected using enhanced chemiluminescence substrate kits (ab133406, Abcam) and visualized with a GE Amersham Imager 600 (AI600; GE Healthcare). Full unedited gel/blot from this study are available in Supplementary Figure S7–9.

### Statistical analysis

The data of 16S rRNA sequencing and intestinal RNA-Seq were analyzed using the tools and methodologies provided by Gene Denovo (Guangzhou, China). Other data was analyzed employing SPSS 22.0 software. The Shapiro – Wilk test assessed data normality, while Levene’s test evaluated variance homogeneity. Data that followed a normal distribution were presented as mean ± standard deviation. The student’s t-test was used for pairwise group comparisons. For analyses involving three or more groups, a one-way analysis of variance (ANOVA) was executed, with Bonferroni’s post hoc test applied for data with normal distribution. For data not fitting a normal distribution, the nonparametric Kruskal – Wallis test was used. The MWM test’s escape latency was analyzed using repeated measure two-way ANOVA, considering “day” as the within-subject factor and “group” as the between-subject factor. Correlations between different experiments were determined using Spearman correlation analysis conducted in R (version 3.5.3). *P*  < 0.05 as statistically significant.

## Results

### HI insult induced long-term cognitive impairments and gut microbial dysbiosis in neonatal rats

The classical Rice – Vannucci modeling approach was performed to construct the neonatal HIBD model in postnatal day 7 rats. Cognitive behavior was assessed using the MWM, NOR, and Y-maze tests between days 28 and 37 following HI insult (Figure 1(a)). The results of MWM (Figure 1(b)) demonstrated that HI insult did not cause motor dysfunction in rats, as evidenced by the lack of statistically significant differences in swimming speed between the sham group and the HI group over five consecutive days post-HI insult (Figure 1(c)). However, rats in the HI group demonstrated a significantly increased escape latency, and a substantial reduction in both number of platform crossing and duration of stay in the target quadrant (Figure 1(d–g)). Likewise, the NOR (Figure 1(h,i)) and Y-Maze (Figure 1(j,k)) evaluations revealed a notable decrease in the recognition index and spontaneous alternation rate in the HI group. These findings collectively corroborate that HI insult can elicit long-term cognitive impairments in neonatal rats, aligning with the outcomes observed in the infants with neonatal HIBD.

**Figure 2.**
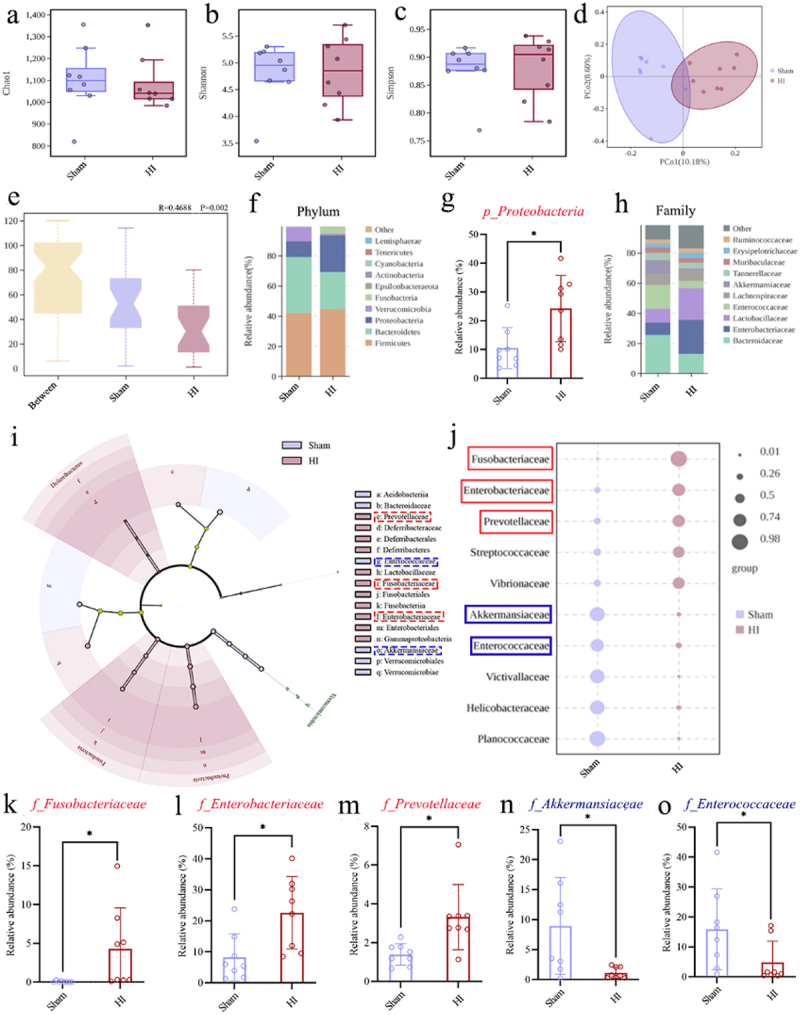
HI insult induced gut microbial dysbiosis in neonatal rats. a-c Analysis of the α-diversity of gut microbiota in the feces of two neonatal rat groups by Chao 1, Shannon, Simpson analysis. d Principal coordinate analysis (PCoA) was employed to calculate the Bray-Curtis distance matrix to analyze β-diversity of gut microbiota in the feces of two neonatal rat groups. e Analysis of similarities (ANOSIM) revealed significant differences in gut microbiota composition between the fecal samples of two neonatal rat groups. f Average relative abundances of gut microbiota at the phylum levels. g the abundance values of *p_Proteobacteria*. h Average relative abundances of gut microbiota at the family levels. i Linear discriminant analysis effect size (LEfSe) was used to identified bacterial species exhibiting significant differences at all taxonomic levels of the gut microbiota of two group’s fecal samples. j Indicator species analysis was used to identified bacterial species exhibiting significant differences at family levels of the gut microbiota of two group’s fecal samples. k-o the abundance values of *f_Fusobacteriaceae*, *f_Enterobacteriaceae*, *f_Prevotellaceae*, *f_Akkermansiaceae*, and *f_Enterococcaceae*. HI, hypoxic-ischemic; *n* = 8, per group; *p < 0.05.

To further determine the roles of gut microbial factors in the HI insult-induced cognitive impairments, the 16S rRNA gene sequencing was used to analyze the composition of the gut microbiota in the fecal samples of the two groups on the 3rd day following HI insult (Figure 1(a)). The results of α-diversity analysis (Figure 2(a–c)) indicated no statistically significant differences in the abundances (Chao1) and diversities (Shannon and Simpson) of gut microbiota in the fecal samples from the two groups of rats. However, when analyzing β-diversity of the gut microbiota in feces using Principal Coordinates Analysis (PCoA), the results revealed a distinct separation of microbial community structures between two groups (Figure 2(d)). In the Analysis of Similarities (ANOSIM), we found that a higher value in the “Between” category compared to within-group (Sham and HI group) values indicates that the between-group differences are significantly larger than within-group differences, with *R* = 0.4688 and *p* = 0.002 (Figure 2(e)). These results suggest that HI insult can causes severe gut microbial dysbiosis in neonatal rats. At the phylum level, the relative abundance of the *p_Proteobacteria* increased in the HI group, while the relative abundance of the phylum *p_Bacteroidetes* decreased (Figure 2(f,g)). At the family level, we observed dysbiosis in multiple gut microbiota between the two groups (Figure 2(h)). Additionally, using Linear discriminant analysis Effect Size (LEfSe) and indicator species analysis at the family level (Figure 2(i,j)), we identified significant microbiota differences between the groups, with *f_Fusobacteriaceae*, *f_Enterobacteriaceae*, *f_Prevotellaceae, f_Streptococcaceae, and f_Vibrionaceae* enriched in the HI group rats, while *f_Akkermansiaceae*, *f_Enterococcaceae, f_Victivallaceae, f_Helicobacteraceae, and f_Planococcaceae* were enriched in the sham group rats. Available studies^41–45^ have shown that *f_Fusobacteriaceae*, *f_Enterobacteriaceae*, and *f_Prevotellaceae* are linked to inflammatory responses, whereas *f_Akkermansiaceae* and *f_Enterococcaceae* exhibit anti-inflammatory effects. Thus, we determined the differences in the relative abundances of these five bacteria between the sham and HI groups and found that the relative abundances of *f_Fusobacteriaceae*, *f_Enterobacteriaceae*, and *f_Prevotellaceae* in the gut microbiota were significantly elevated in the HI group compared with the Sham group (Figure 2(k–m)). Conversely, the abundances of *f_Akkermansiaceae* and *f_Enterococcaceae* were notably reduced in the HI group (Figure 2(n,o)). All of these findings suggest that HI insult induces gut microbial dysbiosis in the early-stage in the neonatal HIBD rats.

**Figure 3.**
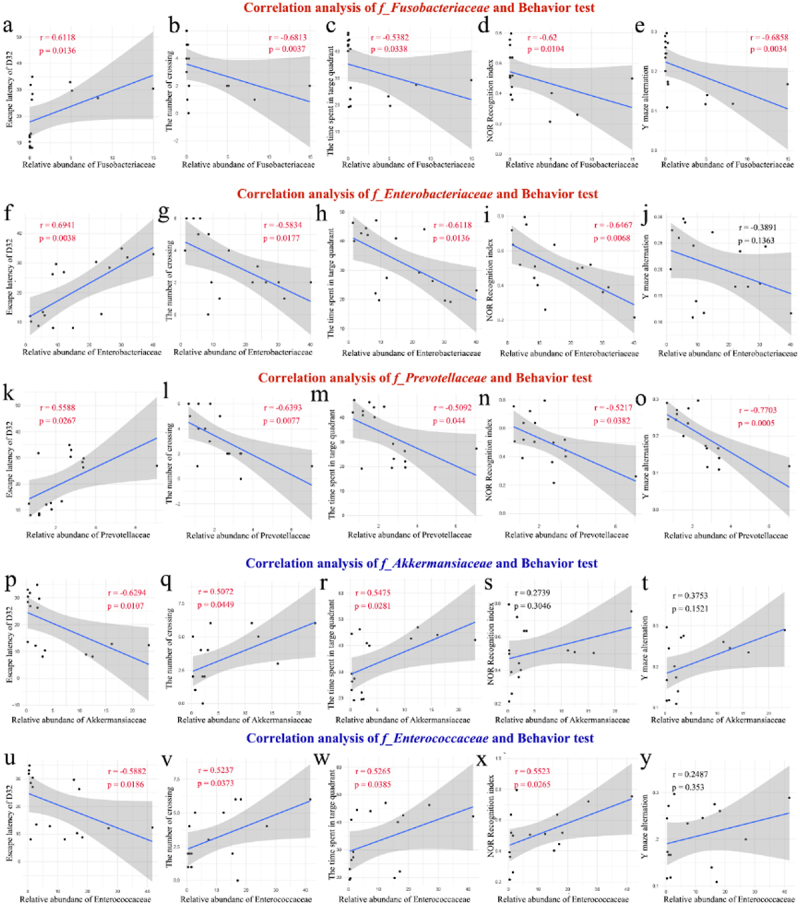
The correlation analyses supported the involvement of the gut microbiota in the development of long-term cognitive impairments in the neonatal HIBD rats. a-y the correlation analysis between the relative abundance of five significantly altered microbial groups (including *f_Fusobacteriaceae*, *f_Enterobacteriaceae*, *f_Prevotellaceae*, *f_Akkermansiaceae*, and *f_Enterococcaceae*) and relevant index data from cognitive behavior tests (including escape latency of D32, the time of crossing, the time spent in target quadrant, recognition index, and Y maze alternation). HIBD, hypoxic-ischemic brain damage; HI, hypoxic-ischemic; D32, the 32 days after HI insult; *n* = 16 in the correlation analysis.

To better explore the correlation between gut microbial dysbiosis and cognitive impairments caused by HI insult, the correlation analyses between the relative abundance of five significantly altered gut microbial families screened from the 16S rRNA sequencing data and relevant index data from cognitive behavior tests (including escape latency of D32, the time of crossing, the time spent in target quadrant, recognition index, and Y maze alternation) were conducted. The results demonstrated a significant association between increased relative abundance of *f_Fusobacteriaceae*, *f_Enterobacteriaceae*, and *f_Prevotellaceae* and more severe cognitive impairments, whereas a decreased relative abundance of *f_Akkermansiaceae* and *f_Enterococcaceae* correlated with more severe cognitive impairments (Figure 3). The correlation results underscore the role of gut microbiota in the onset of long-term cognitive impairments following HI insult in the neonatal rats.

### HI insult induced intestinal dysfunction, and increased serum levels of LPS and inflammatory mediators in the neonatal rats

**Figure 4.**
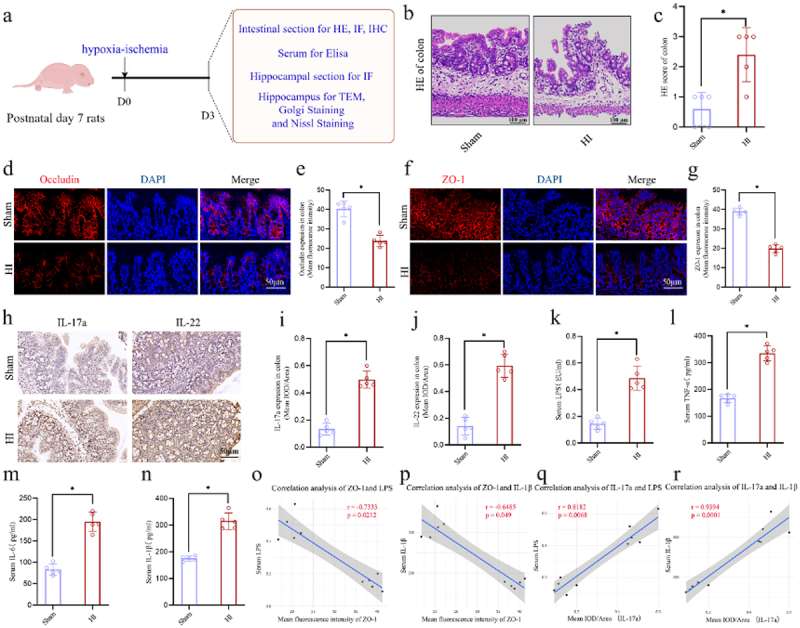
HI insult induced intestinal dysfunction, and increased the serum levels of LPS and inflammatory mediators of neonatal rats. a The experimental flow chart. b, c HE staining of the colons and the pathology scores. d-g the expression levels of the tight junction protein occludin (red) and ZO-1 (red) by the if staining in the colons and mean fluorescence intensity of occludin and ZO-1. h-j The expression levels of the IL-17a and IL-22 in the colons with the IHC staining and the mean IOD/area of these two cytokines. k-n the serum concentrations of LPS, tnf-α, IL-6, and IL-1β. o-r the correlation analysis between the concentrations of pro-inflammatory mediators (including LPS and IL-1β) in the serum and the intestinal function indicators (including the mean fluorescence intensity of ZO-1 and the mean IOD/area of IL-17a in the colon. HI, hypoxic-ischemic; LPS, lipopolysaccharide; HE staining, hematoxylin-eosin staining; if staining, immunofluorescence staining; IHC staining, immunohistochemical staining; IOD, integrated optical density; *n* = 5, per group; *n* = 10 in the correlation analysis; *p < 0.05.

The experimental flow chart exploring the possible role of microbiota-gut-brain axis in the neonatal HIBD is shown in Figure 4(a). The HI group demonstrated significantly worsened histopathological damages and higher histology scores in the colon, compared to the sham group, as evidenced by HE staining (Figure 4(b,c)). The IF staining revealed a significant reduction in the colonic Occludin and ZO-1 expression in the HI group compared to the sham group (Figure 4(d–g)). Moreover, the results of IHC staining showed that, compared to the sham group, the IOD intensity of IL-17a and IL-22 were significantly increased in the HI group (Figure 4(h–j)).

To further investigate whether intestinal dysfunction caused by HI insult affected the concentrations of pro-inflammatory mediators in the blood, an Elisa test was conducted on the serum of neonatal rats on the day 3 following HI insult (Figure 4(a)). The results of Elisa showed that, compared to the sham group, the serum concentrations of LPS, TNF-α, IL-6, and IL-1β were significantly increased in the HI group (Figure 4(k–n)). Additionally, the correlational analysis revealed that the more severe the intestinal dysfunction, the higher the serum concentrations of pro-inflammatory mediators (Figure 4(o–r)). In summary, these results indicate that HI insult can cause the intestinal barrier damage and intestinal inflammation in the neonatal rats. Additionally, intestinal dysfunction induced by HI insult is strongly correlated with increased pro-inflammatory mediators in the circulation.

### Correlation analyses supported the involvement of intestinal dysfunction induced by HI insult in the hippocampal neuroinflammation and synaptic injury

**Figure 5.**
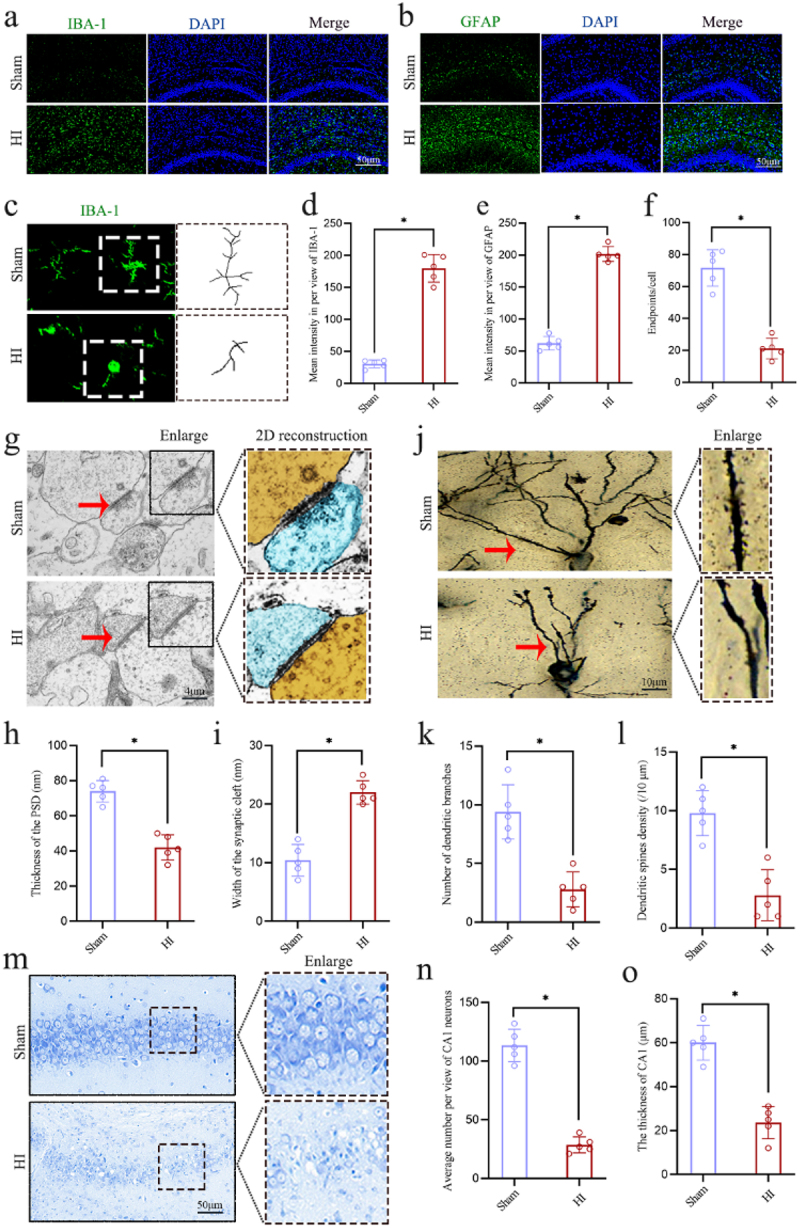
HI insult induced hippocampal neuroinflammation, synaptic injury and neuronal damage in neonatal rats. a, b The expression levels of the microglial marker IBA-1 (green) and astrocyte marker GFAP (green) with the if staining in the hippocampal CA1 region. c Confocal images showed an enlarged view of iba-positive microglia in the hippocampal CA1 region and sholl analyses of microglia imaging were used. d-f the calculated mean intensities of IBA-1 and GFAP in per view, and the number of branches of microglia in the hippocampal CA1 region. g The synaptic ultrastructure changes in the hippocampal CA1 region by TEM. h, i the calculated PSD thickness and synaptic cleft width. **j** the morphology of pyramidal neurons and synaptic structures in the CA1 region of the hippocampus by Golgi staining. k, l the calculated number of dendritic branches and the dendritic spines density. m the neuropathological alterations in the hippocampal CA1 region by Nissl staining. n, o The calculated average number per view of CA1 neurons and the thickness of CA1 were calculated. HI, hypoxic-ischemic; if staining, immunofluorescence staining; TEM, transmission electron microscope; PSD, postsynaptic density; *n* = 5, per group; *p < 0.05.

On the day 3 following HI insult, the hippocampal tissues were collected for test (Figure 4(a)). The results of IF staining showed that HI insult led to significant activation of microglia and astrocytes in the hippocampal CA1 region of neonatal rats (Figure 5(a,b)), manifested by increased number and intensities of both IBA1 and GFAP positive cells (Figure 5(d,e)). Furthermore, the confocal images revealed that HI insult caused microglia in the hippocampal region of neonatal rats to transition from a morphology characterized by small nuclei, long protrusions, and complex shapes to an activated state with enlarged cell bodies, thickened, and reduced branching (Figure 5(c–f)). The TEM was used to observe the ultrastructure of synapses in the hippocampal CA1 region and showed that, in the HI group, pronounced pathological alterations were evident, exemplified by a marked decrease in the thickness of the postsynaptic density (PSD) and an expansion of the synaptic cleft relative to the sham group (Figure 5(g–i)). The Golgi-Cox staining was utilized to determine the impact of HI insult on the morphology of pyramidal neurons and synaptic structures in the hippocampal CA1 region of neonatal rats. The results showed that HI insult resulted in a significant reduction in the density of dendritic spines and dendritic branches in the neonatal rats (Figure 5(j–l)). Additionally, the Nissl staining revealed significant neuropathological alterations in the hippocampal CA1 region in the HI group when compared to the sham group, which included nuclear shrinkage, neuronal loss, and reduced thickness (Figure 5(m–o)). These results indicate that HI insult induces severe hippocampal neuroinflammation, synaptic injury and neuronal damage in the neonatal rats

**Figure 6.**
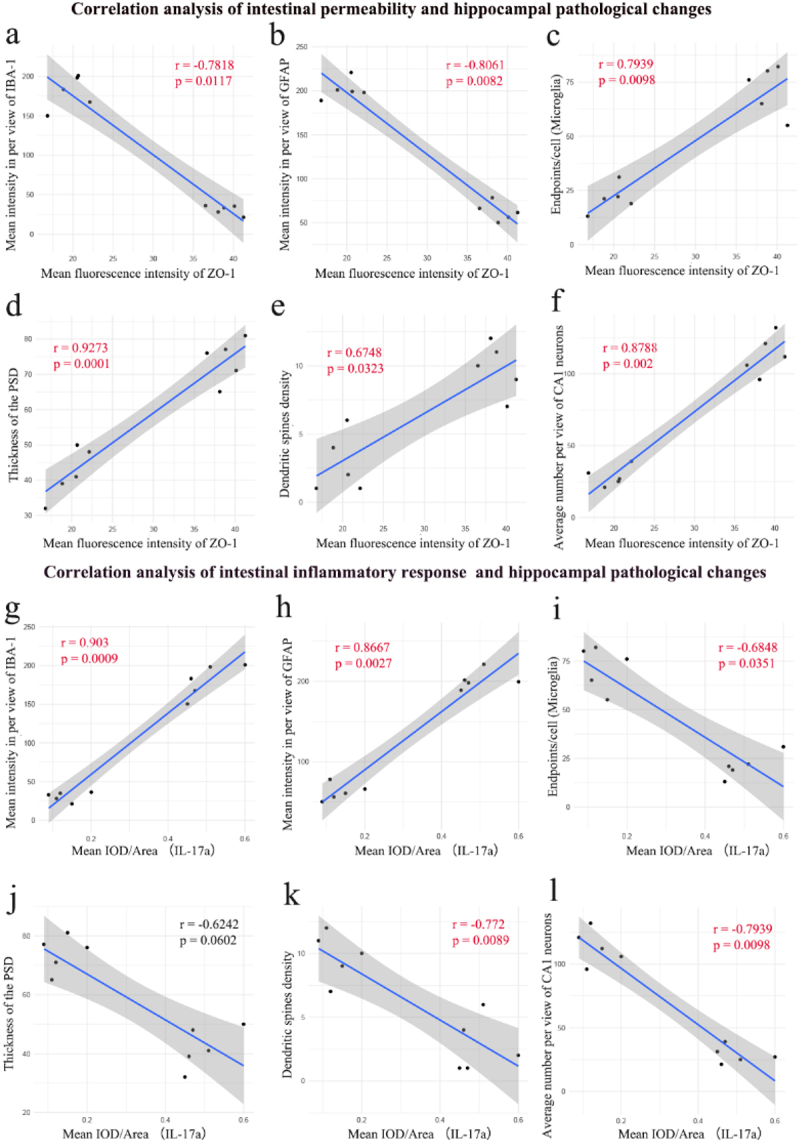
The correlations analyses supported the involvement of the intestinal dysfunction in the hippocampal pathological changes in the neonatal HIBD rats. a-l the correlation analysis between the intestinal function indicators (including the mean fluorescence intensity of ZO-1 and the mean IOD/Area of IL-17a in the colon) and the representative indicators of hippocampal pathological changes (mean intensities of IBA-1 and GFAP per view, endpoints/cell of microglia, thickness of PSD, dendritic spines density, and average number of CA1 neurons per view). Aimed to explore the relation between intestinal function and hippocampal pathological changes induced by HI insult. HIBD, hypoxic-ischemic brain damage; HI, hypoxic-ischemic; PSD, postsynaptic density; IOD, integrated optical density; *n* = 10 in the correlation analysis.

To better explore the correlation between the intestinal dysfunction and the hippocampal pathological changes following HI insult, the additional correlation analyses were conducted to determine the possible relationships between the representative indicators of intestinal dysfunction (mean fluorescence intensity of ZO-1 and mean IOD/Area of IL-17a) and the representative indicators of hippocampal pathological changes (mean intensities of IBA-1 and GFAP per view, endpoints/cell of microglia, thickness of PSD, dendritic spines density, and average number of CA1 neurons per view). The results revealed significant negative correlations between the mean fluorescence intensity of ZO-1 in the colon and the mean intensities of both IBA-1 (*r* = −0.7817, *p* = 0.0117) and GFAP (*r* = −0.8061, *p* = 0.0082) per view in the hippocampal CA1 region (Figure 6(a,b)). Conversely, the mean fluorescence intensity of ZO-1 in the colon was significantly positively correlated with the endpoints per cell of microglia (*r* = 0.7939, *p* = 0.0098), PSD thickness (*r* = 0.9273, *p* = 0.0001), dendritic spine density (*r* = 0.6748, *p* = 0.0323), and the average number of neurons per view (*r* = 0.8788, *p* = 0.0002) in the hippocampal CA1 region (Figure 6(c–f)). These findings imply that the more severe the intestinal barrier damage, the more significant the pathological changes of hippocampal damage, including neuroinflammation, synaptic injury, and neuronal damage. Additionally, the results also revealed significant positive correlations between the mean IOD/Area of IL-17a in the colon and the mean intensities of both IBA-1 (*r* = 0.903, *p* = 0.0009) and GFAP (*r* = 0.8667, *p* = 0.0027) per view in the hippocampal CA1 region (Figure 6(g,h)). Conversely, the mean IOD/Area of IL-17a in the colon was significantly negatively correlated with the endpoints per cell of microglia (*r* = −0.6848, *p* = 0.0351), dendritic spine density (*r* = −0.772, *p* = 0.0089), and the average number of neurons per view (*r* = −0.7939, *p* = 0.0098) in the hippocampal CA1 region (Figure 6(i–l)). These findings suggest that the more severe the intestinal inflammatory responses, the more significant the pathological changes of hippocampal damage, including neuroinflammation, synaptic injury, and neuronal damage.

Together, all of these data suggest that the gut microbial dysbiosis following HI insult may exacerbate long-term cognitive impairments by promoting intestinal dysfunction, leading to the entry of gut microbiota-derived LPS and intestine-derived inflammatory mediators into the bloodstream, worsening hippocampal neuroinflammation, synaptic injury, and neuronal damage.

### The effects of FMT treatment on the intestinal function, systemic inflammation, synaptic structure and long-term cognitive function in the neonatal rats

**Figure 7.**
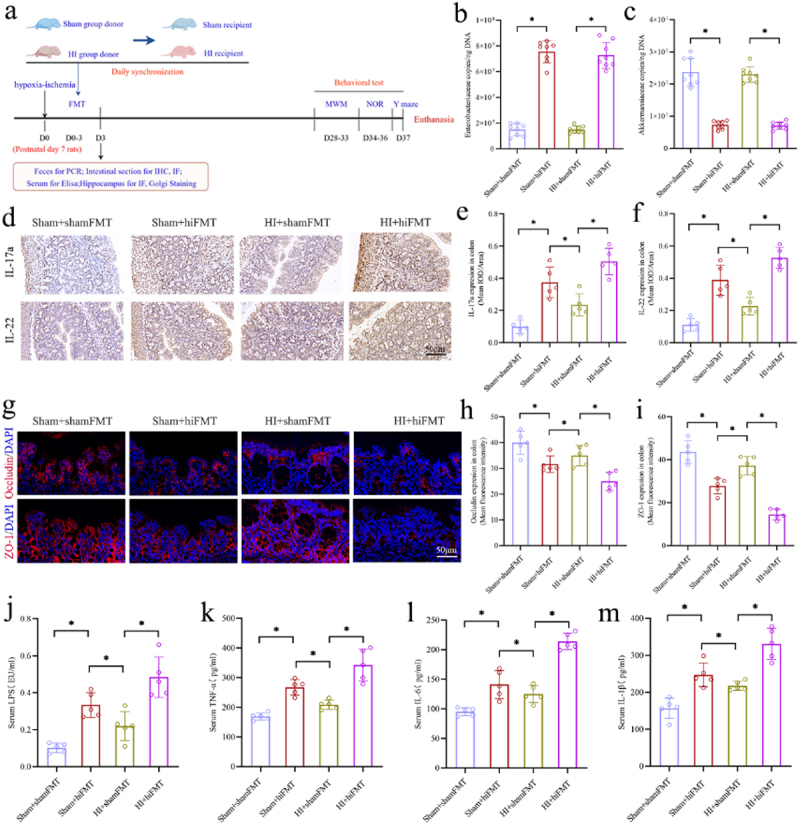
The effects of FMT on the intestinal function and serum inflammation levels in the neonatal rats. a The experimental flow chart. b, c Quantification of *f_Enterobacteriaceae* and *f_Akkermansiaceae* in fecal samples. d-f the expression levels of IL-17a and IL-22 in the colons with the IHC staining and the mean IOD/area of these two cytokines. g-i The expression levels of the tight junction protein occludin (red) and ZO-1 (red) by the if staining in the colons and mean fluorescence intensity of occludin and ZO-1. j-m the serum concentrations of LPS, tnf-α, IL-6, and IL-1β. FMT, fecal microbiota transplantation; HIBD, hypoxic-ischemic brain damage; HI, hypoxic-ischemic; IHC staining, immunohistochemical staining; IOD, integrated optical density; if staining, immunofluorescence staining; LPS, lipopolysaccharide; *n* = 5 or 8, per group; *p < 0.05.

The experimental flow chart to explore the causal relationship between gut microbiota and long-term cognitive impairments induced by HI insult through the FMT is shown in Figure 7(a). Fecal microbiota from sham group and HI group donor rats were transferred to sham group and HI group recipient rats, respectively. The transplantation procedure involved administering FMT once daily from the postnatal 7th day until the 3rd day after HI insult. The samples of feces, colon, serum, and hippocampus were collected for analysis on the day 3 following HI insult, and cognitive behavior was assessed using the MWM, NOR, and Y-maze tests between days 28 and 37 following HI insult. The effect of FMT was assessed through the quantification of specific fecal bacteria using PCR. Two representative bacteria, *f_Enterobacteriaceae* and *f_Akkermansiaceae*, previously identified from 16S rRNA sequencing data, were found to exhibit significant changes following HI insult. Specifically, *Enterobacteriaceae*, identified as harmful bacteria, showed a significant increase, while *f_Akkermansiaceae*, identified as beneficial bacteria, exhibited a significant decrease post-HI insult. The results showed that the content of *Enterobacteriaceae* in the group (Sham+hiFMT, HI+ hiFMT) receiving FMT from HI group donor rats was significantly higher than that in the group (Sham+shamFMT, HI+ shamFMT) receiving FMT from sham group donor rats. Conversely, the content of *f_Akkermansiaceae* in the Sham+hiFMT and HI+ hiFMT groups was significantly lower than that in the Sham+shamFMT and HI+ shamFMT groups (Figure 7(b,c)). These findings suggest that the FMT strategy in this study could effectively replace the original microbiota with the transplanted microbiota. Next, the findings from IHC and IF analysis on colon of neonatal rats demonstrated that, compared to the Sham+shamFMT group, rats in the Sham+hiFMT group exhibited intestinal dysfunction, which was characterized by a significant upregulation of IL-17a and IL-22 expression in colon, and a downregulation of the expression of tight junction-associated proteins occludin and ZO-1. Additionally, the intervention with FMT from the sham group donor rats led to a significant reduction in the expression levels of IL-17a and IL-22 cytokines, as well as an increase in the expression levels of tight junction-associated proteins occludin and ZO-1 in neonatal HIBD rats (HI +shamFMT), compared to those in the HI+hiFMT group (Figure 7(d–i)). The Elisa results showed that the serum levels of LPS, TNF-α, IL-6, and IL-1β in the Sham+hiFMT group were significantly higher than those in the Sham+shamFMT group. However, the serum levels of above pro-inflammatory mediators in the HI+shamFMT group were significantly lower than those in the HI + hiFMT group (Figure 7(j–m)). These findings suggest that gut microbial dysbiosis following HI insult can induce intestinal dysfunction and elevate serum levels of pro-inflammatory mediators in the neonatal rats, and transplanting normal fecal microbiota can effectively ameliorate the intestinal dysfunction and serum inflammation levels in the neonatal HIBD rats. Interestingly, compared to HI+shamFMT group, the Sham+hiFMT group exhibited more severe intestinal dysfunction and higher serum levels of pro-inflammatory mediators (Figure 7(d–m)). These findings suggest that gut microbial dysbiosis following HI insult is a main factor causing intestinal dysfunction and peripheral inflammation in the neonatal rats.

**Figure 8.**
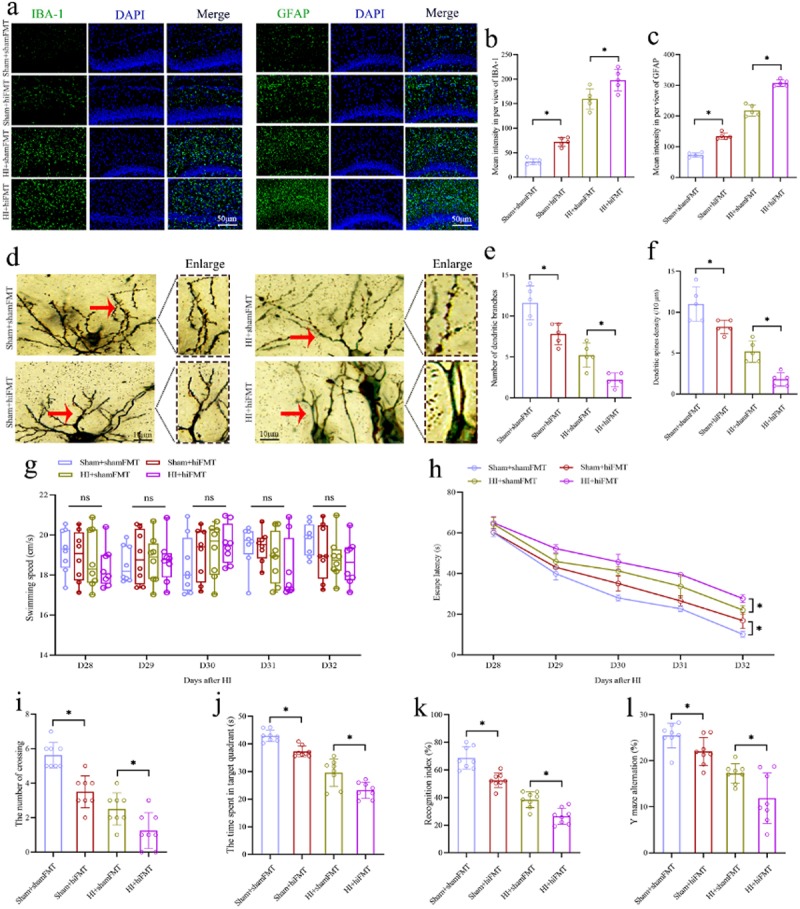
The effects of FMT on the hippocampal neuroinflammation, synaptic structure and long-term cognitive function in the neonatal rats. a The expression levels of microglial marker IBA-1 (green) and astrocyte marker GFAP (green) with the if staining in the hippocampal CA1 region. b, c The mean intensity in per view of IBA-1 and GFAP in the hippocampal CA1 region. d-f The morphology of pyramidal neurons and synaptic structures in the hippocampal CA1 region with the golgi staining and calculated number of dendritic branches and the dendritic spines density. g, h The swimming speed and escape latency of rats in the MWM test from 28 to 32 days after HI insult. i, j The number of crossing the platform and the time spent in the target quadrant of rats in the MWM test on the 33 days after HI insult. k The recognition index in the nor test of rats on the 36 days after HI insult. l The rate of Y maze alternation of rats on the 37 days after HI insult. FMT, fecal microbiota transplantation; HI, hypoxic-ischemic; if staining, immunofluorescence staining; MWM, Morris water maze; NOR, novel object recognition; *n* = 5 or 8, per group; *p < 0.05, ns means no significant.

To determine the effects of FMT on hippocampus and long-term cognitive function in neonatal rats, further experiments were conducted. The results of IF analysis showed a significant increase in the number and intensity of IBA-1 and GFAP-positive cells in the hippocampal CA1 region in the Sham+hiFMT group compared to the Sham+shamFMT group. Additionally, a significant decrease in the number and intensity of IBA-1 and GFAP-positive cells was observed in the hippocampal CA1 region in the rats from the HI+shamFMT group compared to the HI+hiFMT group (Figure 8(a–c)). Subsequently, a notable reduction in the density of dendritic spines and branches in the hippocampal CA1 region was noted in the Sham+hiFMT group compared to the Sham+shamFMT group, as revealed by Golgi staining. Conversely, there was a marked elevation in the density of dendritic spines and branches in the hippocampal CA1 region in the HI+shamFMT group compared to the HI+hiFMT group (Figure 8(d–f)). In the cognitive behavior test, the results of MWM showed that the Sham+hiFMT group demonstrated a significantly increased escape latency and a substantial decrease in both number of platform crossing and duration of stay in the target quadrant compared to the Sham+shamFMT group. Additionally, the HI+shamFMT group demonstrated a significantly decreased escape latency and a substantial increase in both platform crossings and duration of stay in the target quadrant compared to those in the HI+hiFMT group (Figure 8(h–j)). There was no significant difference in swim velocity among these groups (Figure 8(g)), indicating that the above differences are due to differences in cognitive function. Likewise, the results of NOR and Y-Maze revealed a notable decrease in the recognition index and spontaneous alternation rate in the Sham+hiFMT group compared to the Sham+shamFMT group, and a notable increase in the recognition index and spontaneous alternation rate in rats from the HI+shamFMT group compared to the HI+hiFMT group (Figure 8(k,l)). These results indicate that gut microbial dysbiosis following HI insult can induce hippocampal neuroinflammation, and synaptic damage, and long-term cognitive impairments. Moreover, FMT from normal fecal microbiota can mitigate hippocampal neuroinflammation, synaptic injury and long-term cognitive impairments in the neonatal HIBD rats.

In summary, these data confirms that HI insult-induced gut microbial dysbiosis exacerbates long-term cognitive impairments in neonatal rats by promoting intestinal dysfunction, characterized by the activation of intestinal inflammation and disruption of intestinal barrier integrity. This dysregulation of intestinal function ultimately leads to elevated levels of pro-inflammatory mediators, including TNF-α, IL-6, IL-1β, and gut microbiota-derived LPS in the bloodstream, thereby promoting hippocampal neuroinflammation and synaptic damage. Moreover, our data further indicates that the transplantation of normal fecal microbiota can effectively mitigate the aforementioned pathological changes, ultimately alleviating long-term cognitive impairments in the neonatal HIBD rats.

### Oral DEX improved intestinal and long-term cognitive function in the neonatal HIBD rats by partially dependent on gut microbiota

**Figure 9.**
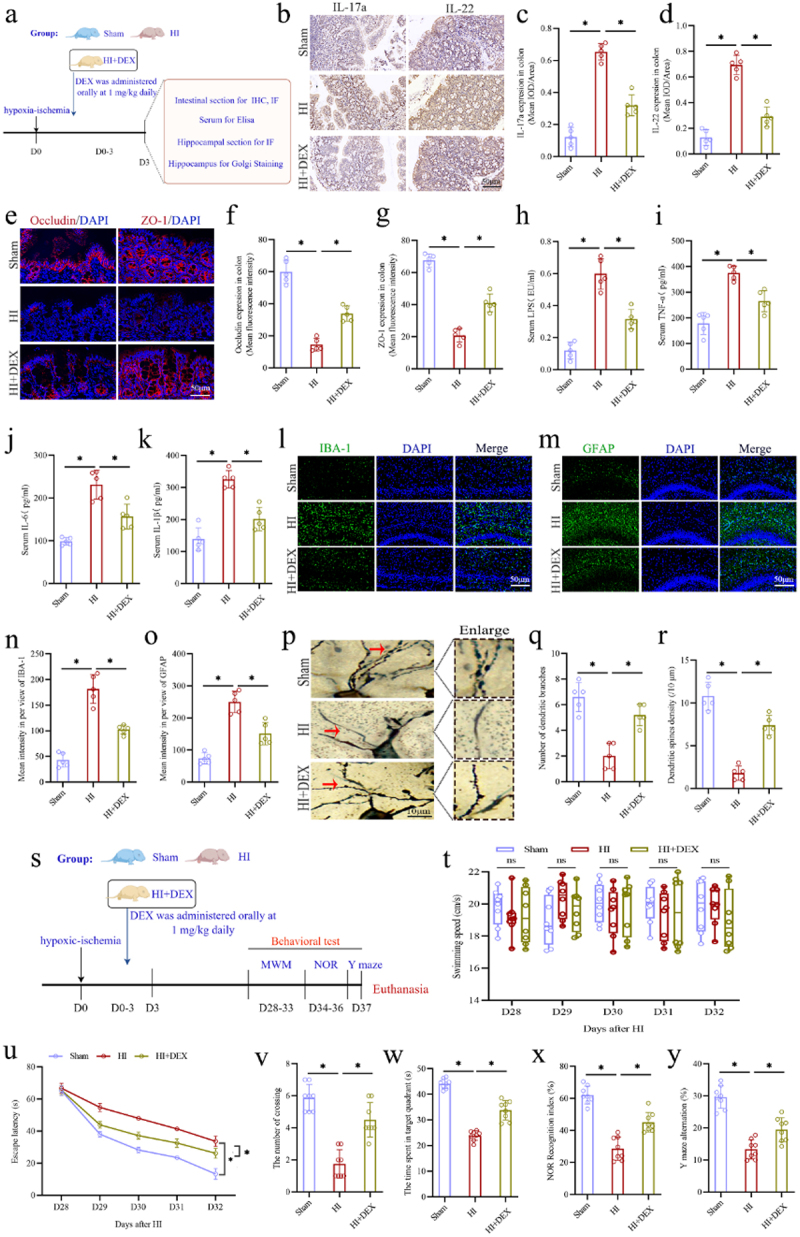
Oral anti-inflammatory agent DEX treatment improved intestinal dysfunction, reduced systemic inflammation, and alleviated synaptic and cognitive impairments in neonatal HIBD rats. a the experimental flow chart. b-d the expression levels of the IL-17a and IL-22 in the colons with the IHC staining and the mean IOD/area of these two cytokines. e-g The expression levels of the tight junction protein Occludin (red) and ZO-1 (red) by the if staining in the colons and mean fluorescence intensity of Occludin and ZO-1. h-k The serum concentrations of LPS, tnf-α, IL-6, and IL-1β. l, m the expression levels of the microglial marker IBA-1 (green) and astrocyte marker GFAP (green) in the hippocampal CA1 region with the if staining. n, o The mean intensities of IBA-1 and GFAP per view in the CA1 region of hippocampus. p-r the morphology of pyramidal neurons and synaptic structures in the CA1 region of hippocampus with the Golgi staining and calculated number of dendritic branches and the dendritic spines density. s the experimental flow chart. t, u The swimming speed and the escape latency of rats in the MWM test from 28 to 32 days after HI insult. v, w the number of crossing the platform and the time spent in the target quadrant of rats in the MWM test on the 33 days after HI insult. x the recognition index in nor test of rats on the 36 days after HI insult. y the rate of Y maze alternation of rats on the 37 days after HI insult. DEX, dexamethasone; HIBD, hypoxic-ischemic brain damage; HI, hypoxic-ischemic; IHC staining, immunohistochemical staining; IOD, integrated optical density; if staining, immunofluorescence staining; LPS, lipopolysaccharide; MWM, Morris water maze; NOR, novel object recognition; *n* = 5 or 8, per group; *p < 0.05, ns means no significant.

The HIBD neonatal rats were subjected to oral administration of DEX daily for a total of four times, from the day of HI insult to 3 days post-insult, and the samples of colon, serum, and hippocampus were collected for analysis on the day 3 following HI insult (Figure 9(a)), as well the cognitive behavior was assessed using the MWM, NOR, and Y-maze tests between days 28 and 37 following HI insult (Figure 9(s)). The results of IHC analysis showed that oral DEX significantly inhibited the expression of IL-17a and IL-22 in the colonic tissues of neonatal HIBD rats, as evidenced by the significant reductions in the intensity of IOD of both cytokines (Figure 9(b–d)). Furthermore, the IF results indicated that oral DEX significantly enhanced the integrity of intestinal barrier, as evidenced by increased expression of tight junction-associated proteins occludin and ZO-1 in the colon of neonatal HIBD rats (Figure 9(e–g)). Additionally, serum levels of LPS, TNF-α, IL-6, and IL-1β in the HI + DEX group were significantly lower than those in the HI group (Figure 9(h–k)). These results suggest that oral DEX significantly suppresses intestinal inflammation and regulates intestinal barrier function, consequently leading to a reduction in circulating pro-inflammatory mediators. To assess the effect of oral DEX on hippocampal pathological changes in the neonatal HIBD rats, further experiments were performed. Analogous to the effects of FMT treatment, the results revealed that oral DEX markedly mitigated hippocampal neuroinflammation, evidenced by significant decreases in both the number and intensities of IBA-1 and GFAP positive cells in the hippocampal CA1 region (Figure 9(l–o)). Additionally, DEX treatment considerably improved the morphology of CA1 pyramidal neurons and their synaptic configurations, notably increasing the densities of dendritic spines and branches (Figure 9(p–r)). In the MWM test, oral DEX treatment resulted in a reduced escape latency (Figure 9(u)) and significant increases in both number of platform crossing (Figure 9(v)) and duration of stay in the target quadrant (Figure 9(w)) relative to the HI group. There was no significant difference in swim velocity among three groups (Figure 9(t)). Likewise, the results of NOR and Y-Maze revealed a notable increase in the recognition index and spontaneous alternation rate in the HI + DEX group compared to the HI group (Figure 9(x,y)). These results suggest that oral DEX treatment can mitigate hippocampal neuroinflammation, synaptic injury and long-term cognitive impairments in the neonatal HIBD rats.

**Figure 10.**
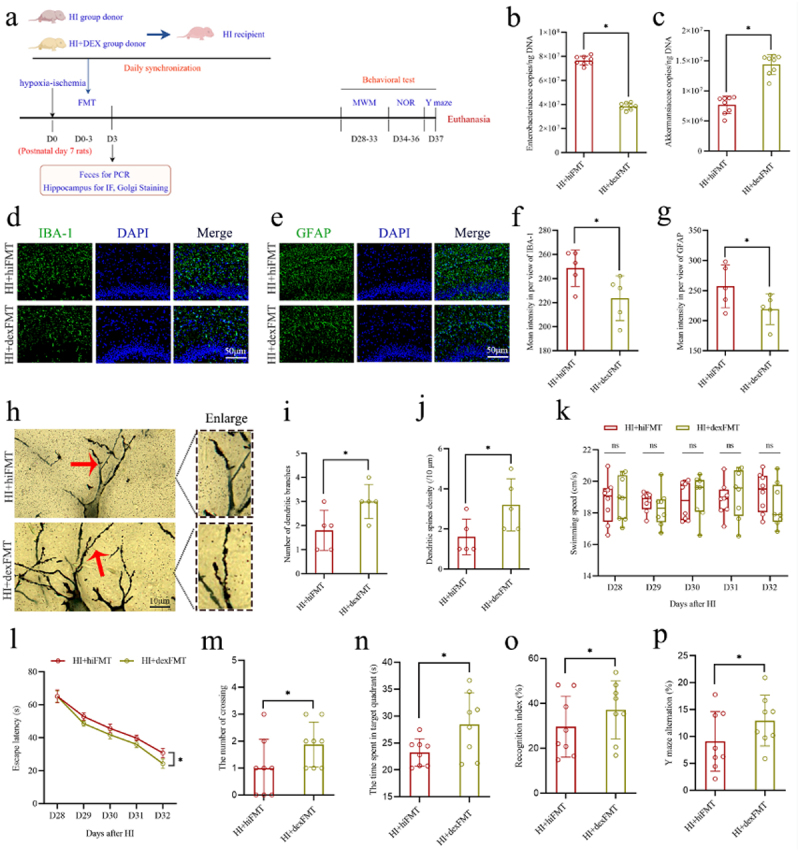
The FMT from the HI+DEX group donors alleviated hippocampal neuroinflammation, synaptic injury, and cognitive impairments in the neonatal HIBD rats. a The experimental flow chart. b, c Quantification of *f_Enterobacteriaceae* and *f_Akkermansiaceae* in fecal samples. d, e The expression levels of the microglial marker IBA-1 (green) and astrocyte marker GFAP (green) in the hippocampal CA1 region with the if staining. f, g The mean intensities of IBA-1 and GFAP per view in the CA1 region of hippocampus. h-j The morphology of pyramidal neurons and synaptic structures in the hippocampal CA1 region with the Golgi staining and calculated number of dendritic branches and the dendritic spines density. k, l The swimming speed and the escape latency of rats in the MWM test from 28 to 32 days after HI insult. m, n the number of crossing the platform and the time spent in the target quadrant of rats in the MWM test on the 33 days after HI insult. o the recognition index in nor test of rats on the 36 days after HI insult. p the rate of Y maze alternation of rats on the 37 days after HI insult. FMT, fecal microbiota transplantation; HI, hypoxic-ischemic; DEX, dexamethasone; if staining, immunofluorescence staining; MWM, Morris water maze; NOR, novel object recognition; *n* = 5 or 8, per group; *p < 0.05, ns means no significant.

To determine whether oral DEX influenced the gut microbiota in the neonatal HIBD rats, the qPCR was used to quantify two specific bacteria, *f_Enterobacteriaceae* and *f_Akkermansiaceae*, in the feces of the Sham group, HI group, and HI+DEX group rats on the 3rd day following HI insult. The results showed that the content of *f_Enterobacteriaceae* in the HI+DEX group was significantly lower than that in the HI group. Conversely, the content of *f_Akkermansiaceae* in the HI+DEX groups was significantly higher than that in the HI groups (Figure s3a, b). Then, a newly designed FMT strategy was designed to verify whether ameliorating gut microbial dysbiosis post-HI insult was a key factor through which oral DEX treatment exerted anti-inflammatory and neuroprotective effects. The fecal microbiota from HI group and HI+DEX group donor rats were transplanted to HI group recipient rats. The transplantation procedure involved administering FMT once daily from the postnatal 7th day until the 3rd day after HI insult. The samples of feces and hippocampus were collected for analysis on the day 3 following HI insult, and cognitive behavior was assessed using the MWM, NOR, and Y-maze tests between days 28 and 37 following HI insult (Figure 10(a)). The results showed that FMT from the HI+DEX group (HI+dexFMT) resulted in a significant decrease in *f_Enterobacteriaceae* content and a significant increase in *f_Akkermansiaceae* content compared to those receiving transplantation from the HI group (HI+hiFMT) (Figure 10(b,c)), suggesting the efficacy of FMT. The results of IF analysis showed significantly decreased number and intensity of IBA-1 and GFAP-positive cells in the hippocampal CA1 region in the HI+dexFMT group compared to the HI+hiFMT group (Figure 10(d–g)). Furthermore, the increased density of dendritic spines and dendritic branches in the hippocampal CA1 region were observed in the HI+dexFMT group compared to the HI+hiFMT group (Figure 10(h–j)). In the cognitive behavior tests, the results of MWM showed that the HI+dexFMT group demonstrated a decreased escape latency and an increase in both number of platform crossing and duration of stay in the target quadrant compared to the HI+hiFMT group (Figure 10(l–n)). There was no significant difference in swim velocity between two groups (Figure 10(k)). Likewise, the results of NOR and Y-Maze revealed a notable increase in the recognition index and spontaneous alternation rate in the HI+dexFMT group compared to the HI+hiFMT group (Figure 10(o,p)). These findings confirm that ameliorating gut microbial dysbiosis following HI insult is one of the key mediators through which DEX treatment exerts its anti-inflammatory and neuroprotective effects.

### The activation of intestinal LPS/TLR4 signaling pathway induced intestinal dysfunction and systemic inflammation, and exacerbated synaptic and cognitive impairments in the neonatal HIBD rats

**Figure 11.**
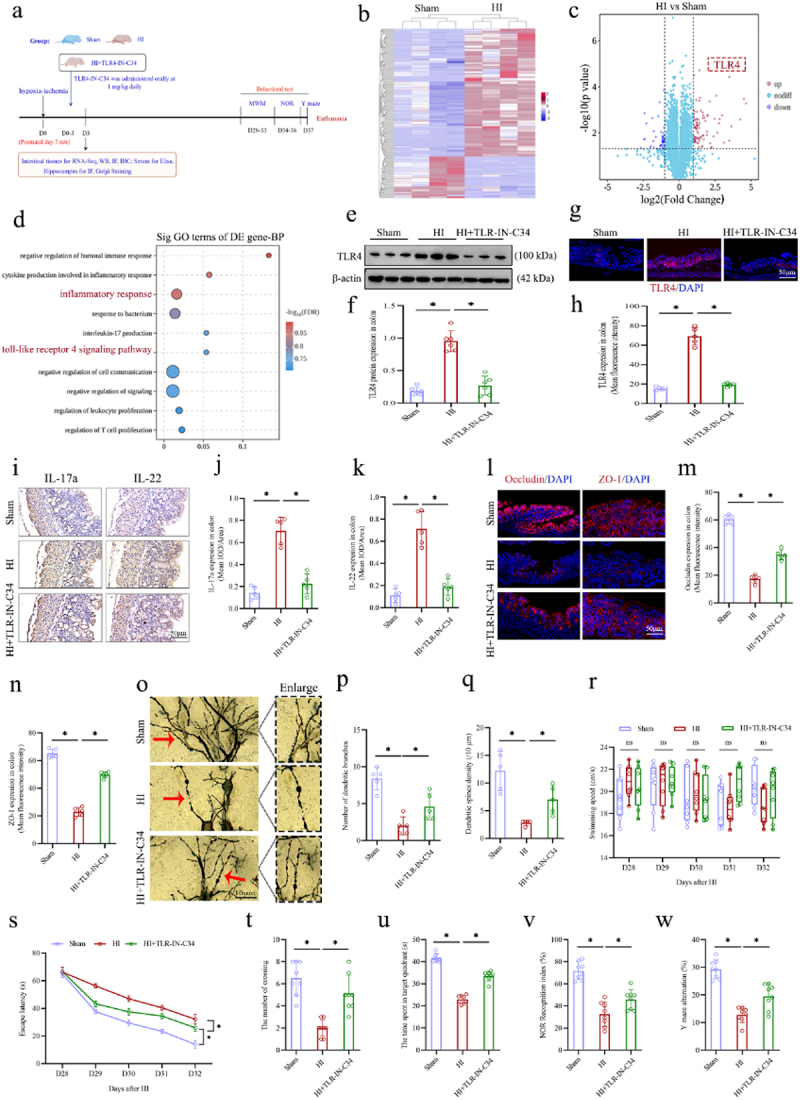
Oral TLR4-IN-C34 treatment improved intestinal dysfunction and alleviated synaptic and cognitive impairments in the neonatal HIBD rats. a the experimental flow chart. b the cluster heat map showing differentially expressed RNA between the sham and HI groups (*n* = 4). c the volcano plots displayed the DEGs as red dots (upregulated) and blue dots (downregulated). d significantly upregulating DEGs were categorized into classes based on GO enrichment terms. e-h the expression levels of TLR4 of colon by WB and IF analyses. i-k the expression levels of the IL-17a and IL-22 in the colons with the IHC staining and the mean IOD/area of these two cytokines. l-n the expression levels of the tight junction protein Occludin (red) and ZO-1 (red) by the if staining in the colons and mean fluorescence intensity of Occludin and ZO-1. o-q the morphology of pyramidal neurons and synaptic structures in the hippocampal CA1 region with the Golgi staining and calculated number of dendritic branches and the dendritic spines density. r, s The swimming speed and the escape latency of rats in the MWM test from 28 to 32 days after HI insult. t, u The number of crossing the platform and the time spent in the target quadrant of rats in the MWM test on the 33 days after HI insult. v the recognition index in nor test of rats on the 36 days after HI insult. w the rate of Y maze alternation of rats on the 37 days after HI insult. TLR4, toll-like receptor 4; HIBD, hypoxic-ischemic brain damage; GO, gene ontology; DEGs, differentially expressed genes; HI, hypoxic-ischemic; WB, western blot; IF, immunofluorescence; IHC staining, immunohistochemical staining; IOD, integrated optical density; if staining, immunofluorescence staining; MWM, Morris water maze; NOR, novel object recognition; *n* = 4, 5, 6 or 8, per group; *p < 0.05, ns means no significant.

The experimental flow chart to further explore the potential molecular mechanisms of intestinal inflammation following HI insult was shown in Figure 11(a). Firstly, the RNA-Seq analysis on the intestinal tissues from the Sham and HI groups was performed. Then, the HIBD neonatal rats were subjected to oral administration of TLR4 specific inhibitor TLR4-IN-C34 for a total of four times, from the day of HI insult to 3 days post-insult, and the samples of colon, serum, and hippocampus were collected for analysis on the day 3 following HI insult, as well as cognitive behavior was assessed using the MWM, NOR, and Y-maze tests between days 28 and 37 following HI insult. The RNA-Seq data showed that genes in the intestinal tissues significantly changed after HI insult (Figure 11(b)), with a notable increase in TLR4 (Figure 11(c)). Additionally, the results of Western blot (WB) and IF staining also showed that the expression of TLR4 in intestine was significantly increased following HI insult (Figure 11(e–h)). This corresponds with above findings about abnormally elevated LPS in neonatal HIBD rats. The analysis of gene ontology (GO) enrichment demonstrated that the upregulating differentially expressed genes (DEGs) were primarily associated with inflammatory responses and TLR4 signaling pathway (Figure 11(d)). Moreover, additional experiments were conducted to examine the fecal LPS levels between the Sham and HI groups. The result indicated that fecal LPS levels were significantly elevated in the HI group compared to the Sham group (Figure S4). These findings suggest that intestinal LPS/TLR4 signaling pathway may be the potential molecular mechanism mediating intestinal dysfunction, exacerbating systemic inflammation, and worsening synaptic and cognitive impairments following HI insult.

To further examine the role of intestinal LPS/TLR signaling in the microbiota-gut-brain axis mechanisms underlying neonatal HIBD, orally TLR4-IN-C34 was used. The results of WB and IF showed that oral TLR4-IN-C34 significantly inhibited the expression of intestinal TLR4 (Figure 11(e–h)). Furthermore, the results of IHC analysis showed that oral TLR4-IN-C34 also significantly inhibited the expressions of IL-17a and IL-22 in the colonic tissues of neonatal HIBD rats, as evidenced by the significant reduction in the intensity of IOD of both cytokines (Figure 11(i–k)). The IF results indicated that oral TLR4-IN-C34 significantly enhanced the integrity of intestinal barrier, as evidenced by increasing the expression of tight junction-associated proteins occludin and ZO-1 in the colons of neonatal HIBD rats (Figure 11(l–n)). In addition, our findings revealed that oral TLR4-IN-C34 also markedly mitigated systemic inflammation, evidenced by significantly decreased serum levels of LPS, TNF-α, IL-6, and IL-1β (Figure S5a-d), as well as the number and intensity of IBA-1 and GFAP positive cells in the hippocampal CA1 region (Figure S5e-g). Most importantly, our findings revealed that oral TLR4-IN-C34 improved the morphology of CA1 pyramidal neurons and their synaptic configurations, notably increasing the density of dendritic spines and branches (Figure 11(o–q)). In the cognitive behavioral tests, the MWM test showed that oral TLR4-IN-C34 treatment resulted in evidently reduced escape latency, and significant increases in both number of platform crossing and duration of stay in the target quadrant relative to the HI group (Figure 11(s–u)). However, there was also no significant difference in swim velocity among three groups (Figure 11(r)). Likewise, the results of NOR and Y-Maze revealed that the recognition index and spontaneous alternation rate were significantly increased in the HI + TLR4-IN-C34 group compared to the HI group (Figure 11(v,w)). To validate whether intestinal dysfunction induced by gut microbial dysbiosis following HI insult was related to the intestinal LPS/TLR4 signaling pathway, further experiments were performed. The results of WB and IF showed that the FMT from sham group rats significantly inhibited the expression of intestinal TLR4 in neonatal HIBD rats (Figure s6a-d). Likewise, oral DEX treatment could also significantly inhibit the expression of intestinal TLR4 in the neonatal HIBD rats (Figure s6e-h). These findings suggest that gut microbial dysbiosis following HI insult activates the LPS/TLR4 signaling pathway, leading to intestinal inflammation and dysfunction, which exacerbates systemic inflammation and, in turn, worsens synaptic and long-term cognitive impairments. Inhibition of intestinal LPS/TLR4 signaling pathway can exert neuroprotective effects in the neonatal HIBD rats.

## Discussion

The neurological sequelae caused by neonatal HIBD, including long-term cognitive impairments, remain a significant clinical challenge that is difficult to address.^2,46^ Due to the complex pathological factors of neural damage post-HIBD,^47^ there are still no effective clinical intervention measures available. Consequently, the exploration of novel strategies and the provision of theoretical foundations hold crucial clinical significance. Consistent with clinical situations^2,46^ and previous animal studies,^1,48^ this study found that HI insult could cause severe long-term cognitive impairments in the neonatal rats. Increasing evidence highlights the critical role of gut microbiota in regulating brain function and cognitive processes.^49^ Moreover, recent studies have demonstrated alterations in gut microbial composition in both human neonates with HIBD^50^ and HIBD animal models^51^ after HI insults. However, the associations between early gut microbial dysbiosis post-HI insult and subsequent long-term cognitive impairments, as well as the underlying microbiota-gut-brain axis mechanisms, remain inadequately explored. Here, we established a causal relationship between early gut microbiota dysbiosis following HI insult and long-term cognitive impairments induced by HIBD. In the neonatal HIBD rat model, we observed significant changes in the relative abundances of certain key bacteria, which were significantly correlated with the metrics of long-term cognitive function. Further research revealed that gut microbiota dysbiosis led to enhanced intestinal inflammatory responses and aggravated intestinal dysfunction, which were closely associated with systemic inflammation, synaptic damage, and long-term cognitive impairments. Using the FMT, we confirmed the causative role of microbiota-gut-brain axis mechanism in the development of long-term cognitive impairments by the neonatal HIBD, suggesting that early correction of gut microbiota dysbiosis after HI insult may prevent the progression of long-term cognitive impairments. Further investigation into the therapeutic effects of oral DEX treatment in neonatal HIBD rat model revealed that its neuroprotective effect was at least partially dependent on suppression of intestinal inflammation and improvement of gut microbial dysbiosis. Analysis of potential molecular mechanisms indicated that activation of the intestinal LPS/TLR4 signaling was the intrinsic mechanism by which gut microbiota dysbiosis led to neurotoxicity post-HI insult, and further oral TLR4-IN-C34 treatment provided effective neuroprotection through the gut-brain axis mechanism by inhibiting TLR4 expression in intestine. Based on these results, this study established the role of the microbiota-gut-brain axis in regulating intestinal function and neuroinflammation following HI insult and proposed the intestinal LPS/TLR4 signaling as a key mechanistic target in mediating this process, providing new insights for molecular mechanisms and potential strategies of long-term cognitive impairments associated with neonatal HIBB.

Our results clearly displayed significant changes in the gut microbiota of neonatal rats on the 3rd day post-HI insult compared to the sham group. Notable increases in *f_Fusobacteriaceae*, *f_Enterobacteriaceae*, and *f_Prevotellaceae*, along with significant decreases in *f_Akkermansiaceae* and *f_Enterococcaceae*, were observed post-HI insult. Studies indicate that *f_Fusobacteriaceae*, *f_Enterobacteriaceae*, and *f_Prevotellaceae* act as promoters of inflammatory responses in a variety of diseases, such as inflammatory bowel diseases and colorectal cancer, through their secretion of metabolites or immune modulation capabilities.^42–44^ *f_Akkermansiaceae*, a key mucosal resident in the gut, has the capacity to uphold gut barrier integrity and prevents cognitive impairments in sleep-deprived mice.^41^ Additionally, *f_Enterococcaceae* has been reported to have anti-cancer, cholesterol-lowering, and immune-modulatory effects. For example, E. durans M4–5, belonging to one of the genera within *f_Enterococcaceae*, has been found to produce butyrate, a short-chain fatty acid, known to exert significant anti-inflammatory effects and enhance intestinal epithelium integrity.^45^ To determine the role of gut microbial alterations following HI insult in the progression of cognitive impairments, the correlations between the relative abundances of these five microbial groups and cognitive behavioral test scores were analyzed in our study. The findings demonstrated a positive correlation between cognitive impairments severity and the abundances of *f_Fusobacteriaceae*, *f_Enterobacteriaceae*, and *f_Prevotellaceae*, alongside a negative correlation with *f_Akkermansiaceae* and *f_Enterococcaceae*. These data suggest that dysbiosis of the gut microbiota post-HI insult, characterized by the upregulation of harmful gut microbiota associated with inflammation and the downregulation of beneficial gut microbiota with protective effects, may further trigger inflammatory responses, and exacerbate the progression of long-term cognitive impairments via the microbiota-gut-brain axis mechanisms. Furthermore, the identified microbial groups may act as potential biomarkers for the neurocognitive prognosis of HIBD.

It is well-known that the intestine directly interacts with the gut microbiota, which can directly modulate the immune and inflammatory responses of the intestinal tissue.^52^ It has been shown that gut microbiota is involved in regulating intestinal inflammation.^53^ Our preliminary research has confirmed that early changes in the gut microbiota following HI insult may be related to enhanced intestinal inflammatory response. Based on the above, we hypothesized that dysbiosis of the gut microbiota following HI insult induces inflammatory response in intestinal epithelial cells and the intestinal barrier damage. This facilitates the entry of pro-inflammatory mediators, including LPS from the gut microbiota and inflammatory cytokines (TNF-α, IL-6, IL-1β) secreted by epithelial cells, into the brain via the bloodstream, promoting systemic inflammation and participating in mediating neural damage after HIBD. In experiments exploring the microbiota-gut-brain axis mechanisms underlying HIBD, our results demonstrated that HI insult significantly compromises the integrity of the intestinal barrier, intensifies the intestinal inflammatory responses, and elevates the serum levels of LPS, TNF-α, IL-6, and IL-1β in the neonatal rats on the 3rd day following the HI insult. Additionally, the correlation analyses about the relationships between the integrity of the intestinal barrier and the serum levels of pro-inflammatory mediators, and between degree of intestinal inflammatory responses and serum levels of pro-inflammatory mediator indicated that both the decreased integrity of intestinal barrier and increased intestinal inflammation were significantly associated with increased serum levels of pro-inflammatory mediators. Neuroinflammation is a key pathological mechanism of neural damage following HIBD, with the activation of microglia and astrocytes being significant components of neuroinflammation.^47^ Previous studies, including own, have confirmed that inhibiting abnormal activation of microglia and astrocytes can effectively mitigate synaptic damage and neurological deficits post-HIBD.^1,5,27,48,54^ Importantly, the synapses in the hippocampus are crucial for the formation and regulation of cognitive functions.^55^ Excessive neuroinflammatory responses may destroy hippocampal synapses, leading to cognitive impairments.^56^ Additionally, peripheral pro-inflammatory mediators entering the brain can mediate the activation of microglia and astrocytes, thus further amplifying the neuroinflammatory responses.^57^ The blood-brain barrier (BBB) plays a crucial role in maintaining brain homeostasis and protecting against harmful substances and pathogens.^58^ Previous studies^59–61^ have fully demonstrated that HI insult severely damages the BBB, allowing more peripheral harmful substances to penetrate the central nervous system, resulting in central nervous system damage. Similarly, our study observed excessive activation of microglia and astrocytes in the hippocampal CA1 region on the 3rd day after HI insult. Additionally, through TEM, Golgi staining, and Nissl staining, we found the destruction in the synaptic structures of neurons in the hippocampal CA1 region, along with significant abnormalities in neuronal morphology and number. Further application of correlation analysis to assess the relationships between the integrity of the intestinal barrier and hippocampal pathological changes, as well as the degree of intestinal inflammatory responses and hippocampal pathological changes, indicated that the decreased integrity of intestinal barrier and intensification of intestinal inflammation were associated with increased activation of microglia and astrocytes, extensive synaptic damage, and reduced number of neurons in the hippocampal CA1 region. These data strongly suggest that dysbiosis of the gut microbiota following HI insult may promote intestinal dysfunction, leading to increased serum pro-inflammatory mediators crossing the BBB to the brain, thereby promoting hippocampal neuroinflammation and ultimately exacerbating damage to hippocampal synapses and long-term cognitive impairments.

The FMT has been proven to improve the prognosis of various neurological diseases, which also serves as a key method to demonstrate the causal relationship between gut microbiota and disease symptoms.^10,52^ To determine the causal relationship between early gut microbial dysbiosis following HI insult, the speculated microbiota-gut-brain axis mechanisms, and long-term cognitive impairments, we conducted FMT based on the methods of previous studies.^38,39^ In this study, we firstly designed two different FMT strategies. Similar to previous studies^10,52^ in other neurological disease, we found that transplanting the fecal microbiota of sham group rats into neonatal HIBD rats could restore the composition of their gut microbiota, alleviate intestinal inflammation, improve intestinal barrier integrity, reduce serum levels of pro-inflammatory mediators, thereby mitigating hippocampal neuroinflammation, synaptic damage, and long-term cognitive impairments. Conversely, transplanting the fecal microbiota from HIBD rats into sham group recipient rats induced gut microbial dysbiosis, mediated intestinal inflammation, reduced barrier integrity, increased serum pro-inflammatory mediator levels, thereby mediating hippocampal neuroinflammation, synaptic damage, and long-term cognitive impairments. Moreover, our analysis comparing the two FMT strategies revealed that, relative to the HI+shamFMT group, rats in the Sham+hiFMT group exhibited more severe intestinal dysfunction and higher serum levels of pro-inflammatory mediators. These results suggest that gut microbial dysbiosis following HI insult is a significant contributor to intestinal dysfunction and systemic inflammation in neonatal HIBD rats. This observation underscores that early-stage gut microbial dysbiosis has a more profound impact on intestinal function than the HI insult itself, aligning with existing literature on the critical role of gut microbiota in maintaining intestinal health.^52,62^ In terms of brain function, although the HI insult was the primary cause of brain damage, gut microbiota significantly influenced the extent of neural injury. These findings are corroborated by other studies^10–12^ investigating the gut-brain axis and neural injury. Additionally, numerous studies^63–65^ have shown that gut microbiota can influence the permeability of the BBB, with dysbiosis facilitating the translocation of intestinal bacteria-derived pathogens and harmful toxins into the circulatory system, leading to BBB breakdown and neuroinflammation. Our study also showed that hippocampal neuroinflammation, synaptic injury, and cognitive impairments occurred in normal rats receiving FMT from HI-insulted donor rats. These findings imply that the gut microbial dysbiosis following HI insult may contribute to BBB disruption, thereby facilitating the entry of more deleterious circulating substances into the brain and exacerbating neurological damage. Therefore, investigating the impact of gut microbial dysbiosis following HI insult on the function of the BBB will be one of the main directions of our future research. Overall, these data demonstrate that gut microbial dysbiosis following HI insult participates in mediating hippocampal neuroinflammation, synaptic damage, and cognitive impairments induced by HIBD through promoting intestinal dysfunction and increasing serum pro-inflammatory mediator levels; therefore, correcting early gut microbial dysbiosis by FMT may be a potential therapeutic strategy for alleviating the long-term neurological sequelae in children with HIBD.

The observed beneficial effects of FMT on cognitive impairments induced by HI insult have motivated us to explore the potential effects of intervening in intestinal dysfunction within a similar research framework. Excessive intestinal inflammation is a key factor in the impairments of intestinal barrier integrity.^10^ DEX, a conventional glucocorticoid and anti-inflammatory drug, has been reported to alleviate intestinal inflammation and intestinal barrier damage caused by various pathogenic factors through oral administration.^10,18,19^ However, in the neonatal HIBD model, the neuroprotective and neurotoxic effects of DEX remain contentious. Studies^66,67^ have shown that glucocorticoids can influence the vulnerability of fetal and neonatal brains to HI challenges, though the outcomes are inconsistent and vary based on experimental protocols, dosages, timings, animal ages, strains, and species. Additionally, the dose and duration of glucocorticoid treatment appear to be the critical factors that determine whether the effects on the brain are detrimental or beneficial. While prolonged exposure to high levels of glucocorticoids increases neurotoxic effects, a physiological or slightly elevated levels provide neuroprotection against HI challenges.^68^ Several studies^69–71^ have demonstrated that glucocorticoid pretreatment, particularly DEX, yields a neuroprotective outcome in some animal models of neonatal HIBD. Despite the promise of these findings, translating them into clinical practice is still challenging. It is noteworthy that prophylactic interventions for HIBD are impractical due to the low incidence and the acute nature of HI events.^66,72^ Interestingly, available evidence shows that post-HI insult treatment with DEX can be both neurotoxic and neuroprotective.^66,72^ However, the neurotoxic effects are typically associated with administration methods, such as high doses and repeated administrations via intraperitoneal and subcutaneous injections,^73–75^ which result in increased systemic effects and side effects. In this study, we focused on the effects of oral DEX administration on the intestine and enhanced the clinical translatability of this intervention by implementing post-treatment. The oral bioavailability of DEX is reported to range between 70% and 78% in humans.^76^ However, this value may differ in neonatal HIBD rat models. Therefore, the administration method and dosage employed in this study were based on previous study^10^ and our pilot experiments. Our pilot findings demonstrated that an oral dose of 1 mg/kg of DEX administered daily for four consecutive days, commencing from the day of HI insult in neonatal HIBD rat model, was safe and effective, with no significant side effects. Based on the above, our main findings further demonstrated that oral DEX treatment effectively suppressed intestinal inflammation, ameliorated intestinal barrier damage, and reduced systemic inflammation, and alleviated synaptic damage, and improved long-term cognitive impairments in neonatal rats with HIBD. In addition, studies indicate that gut microbiota is a critical regulatory factor for the observed anti-inflammatory effects on intestinal tissues after DEX administration.^20,21^ To determine whether gut microbiota also mediates the neuroprotective effects of oral DEX treatment, we employed FMT for validation. Based on the findings of the above work, our results demonstrated that FMT from donor rats in the HI+DEX group alleviated hippocampal neuroinflammation, synaptic injury, and long-term cognitive impairments in neonatal rats with HIBD. These findings suggest that mitigating gut microbial dysbiosis and intestinal dysfunction following HI insult is a crucial mechanism through which oral DEX treatment exerts its neuroprotective effects.

To explore the potential molecular mechanisms of the microbiota-gut-brain axis underlying neonatal rats with HIBD, the high-throughput RNA-Seq was conducted to observe the genes and molecular pathways involved in intestinal inflammation following HI insult. Our analysis revealed a significant increase in TLR4 expression in the intestines of neonatal HIBD rats. The TLR4 is a specific receptor for LPS and plays a crucial role in inflammation mediated by the microbiota-gut-brain axis mechanisms.^77^ A major producer of LPS in the gut microbiota is Gram-negative bacteria, with *f_Enterobacteriaceae* being a key example.^78^ A recent study suggested the excessive proliferation of *f_Enterobacteriaceae* exacerbates systemic inflammation and worsens cerebral infarction via the LPS/TLR4 pathway.^79^ In our study, we found a significant increase in the abundance of *f_Enterobacteriaceae* and the levels of LPS in the feces of neonatal rats after HI insult. Based on the findings of the above work, we further speculated that the LPS/TLR4 signaling pathway in intestinal tissue was a key intrinsic molecular mechanism of the microbiota-gut-brain axis in neonatal HIBD rats. Therefore, effectively inhibiting TLR4 expression in intestinal tissue is crucial for validating this hypothesis. Previous studies^22–24^ have demonstrated that TLR4-IN-C34 is an orally active, selective TLR4 inhibitor that can suppress TLR4 signaling activation through oral administration, improving intestinal and systemic inflammation in necrotizing enterocolitis, ulcerative colitis and other intestinal disease models, suggesting its therapeutic potential for intestinal inflammation. Therefore, TLR4-IN-C34 was selected in this study. The administration method and dosage employed in this study were based on previous study and our pilot experiments. Furthermore, our results showed that treatment with the orally active TLR4-specific inhibitor TLR4-IN-C34 significantly improved intestinal dysfunction, systemic inflammation, and brain function damage in neonatal HIBD rats. Through reviewing the literature,^80–82^ we found that TAK-242 is also a widely used selective TLR4 inhibitor, but its administration in neonatal HIBD or other disease animal models is mostly through intraperitoneal injection, rather than the oral administration used in our study, a more clinically applicable method. However, inhibiting TLR4 through intraperitoneal injection of TAK-242 can also improve effectively the gut microbiota. This provides a new interventional method of TLR4 signaling pathway in our future researches of neonatal HIBD. Additionally, both FMT and oral DEX treatment also significantly inhibited the intestinal LPS/TLR4 signaling pathway. These results suggest that activation of the intestinal LPS/TLR4 signaling pathway is a potential molecular mechanism for the involvement of microbiota-gut-brain axis in the long-term cognitive impairments associated with the neonatal HIBD.

Our study has some limitations that deserve attention. Firstly, this study was conducted in rats, as many factors such as diet that can alter the gut microbiota could be well controlled. Therefore, the five specific gut microbial biomarkers identified from our study need further validation in a large cohort of children with HIBD before they can be applied to humans. Secondly, although this study did not use germ-free pups for FMT intervention, according to methods specified in previous studies,^14,38,39^ a combined antibiotic regimen was administered to the dams producing the recipient pups in order to maximize the elimination of the original gut microbiota of the recipient pups, and applied an effective FMT regimen to the recipient pups. Additionally, after completing the FMT, the qPCR was further utilized to quantify specific bacterial groups identified from our 16S rRNA data to assess the effectiveness of the FMT. These measures minimized the impact of this issue on our research conclusions. Finally, oral DEX has the opportunity to exert a direct protective effect on the brain through the first-pass effect. However, this study detected the intestinal LPS/TLR4 signaling pathway and intestinal function in the neonatal HIBD rats treated with oral DEX, and further verified through the FMT that oral DEX treatment exerted neuroprotective effects on the neonatal HIBD rats at least partially by improving gut microbial dysbiosis and intestinal dysfunction.

## Conclusion

**Figure 12.**
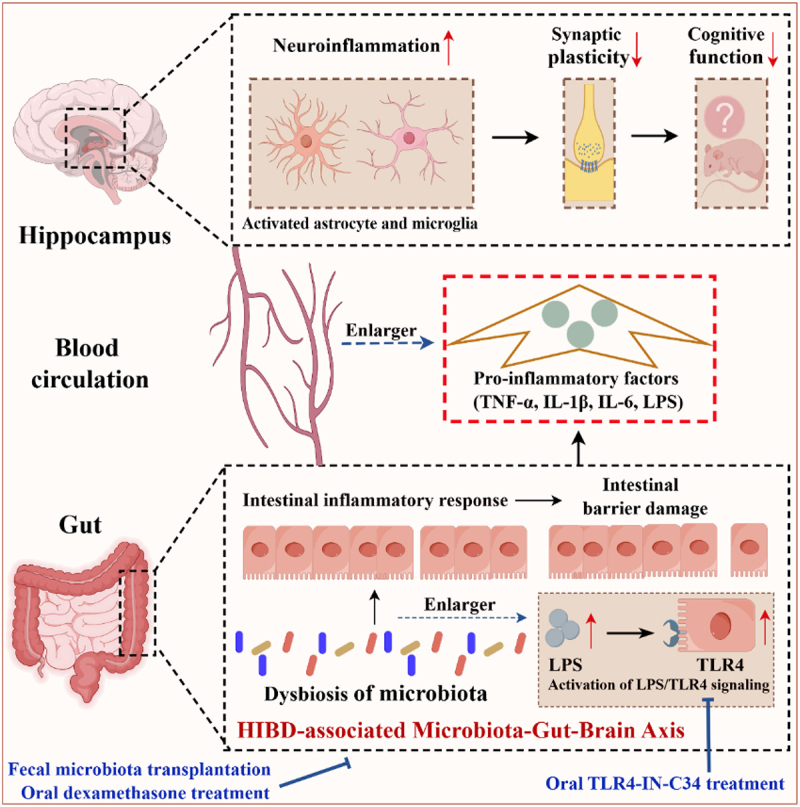
Schematic diagram of the findings from this study. The gut microbial dysbiosis following the HI insult activates LPS/TLR4 signaling pathway, leading to intestinal inflammation and intestinal barrier damage. This intestinal dysfunction can elevate serum levels of pro-inflammatory mediators, including LPS from gut microbiota and tnf-α, IL-6, and IL-1β secreted by intestinal epithelial cells, thereby activating microglia and astrocytes in the hippocampus via the microbiota-gut-brain axis. Consequently, this process can exacerbate hippocampal neuroinflammation, synaptic damage, and long-term cognitive impairments induced by HIBD. Rectifying the gut microbial dysbiosis by the FMT or oral DEX can counteract these detrimental effects and alleviate long-term cognitive impairments caused by neonatal HIBD. Moreover, oral TLR-IN-C34 treatment also can exert neuroprotective effect by inhibiting the intestinal LPS/TLR4 signaling pathway. HI, hypoxic-ischemic insult; LPS, lipopolysaccharide; TLR4, toll-like receptor 4; HIBD, hypoxic-ischemic brain damage; FMT, fecal microbiota transplantation; DEX, dexamethasone; by Figdraw.

In summary, our study suggests that early-stage dysbiosis of the gut microbiota following HI insult can activate the intestinal LPS/TLR4 signaling pathway, leading to excessive intestinal inflammatory responses and intestinal barrier damage. This promotes systemic inflammation, and ultimately worsens hippocampal synaptic damage and long-term cognitive impairments in the neonatal HIBD rats. Transplantation of healthy gut microbiota and oral anti-inflammatory agent DEX can effectively counteract these detrimental effects and alleviate long-term cognitive impairments caused by neonatal HIBD. Moreover, oral administration of TLR-IN-C34 can exert neuroprotective effect by inhibiting the intestinal LPS/TLR4 signaling pathway in the neonatal HIBD rats (Figure 12). Therefore, early correction of gut microbial dysbiosis and intestinal dysfunction may be the potential interventions to alleviate long-term cognitive impairments in children suffering from neonatal HIBD.

## Supplementary Material

Supplementary Information R3.docx

## Data Availability

The 16S rRNA gene sequencing raw data that support the findings of this study are available in https://www.ncbi.nlm.nih.gov/. The BioProject ID in Sequence Read Archive (SRA) of NCBI is PRJNA1115098. The submission name is: Gut microbial dysbiosis exacerbates long-term cognitive impairments by promoting intestinal dysfunction and neuroinflammation following neonatal hypoxic ischemia. The RNA sequencing raw data of intestinal tissues that support the findings of this study have been deposited into CNGB Sequence Archive (CNSA) of China National GeneBank DataBase (CNGBdb) with accession number CNP0006043 (https://db.cngb.org/search/project/CNP0006043). The other original contributions presented in the study are included in the article/Supplementary Material, further inquiries can be directed to the corresponding author.

## Abbreviations

HIBD: Hypoxic-ischemic brain damage
HI: Hypoxic-ischemic
LPS: Lipopolysaccharides
FMT: Fecal microbiota transplantation
DEX: Dexamethasone
MRI: Magnetic resonance imaging
PD: Parkinson’s disease
AD: Alzheimer’s disease
PND: Postoperative neurocognitive disorders
TNF-α: Tumor necrosis factor-alpha
IL-6: Interleukin-6
IL-1β: Interleukin-1beta
SD: Sprague Dawley
MWM: Morris water maze
NOR: Novel object recognition
HE: Hematoxylin and Eosin
IF: Immunofluorescence
IHC: Immunohistochemistry
ELISA: Enzyme-linked immunosorbent assay
TEM: Transmission electron microscopy
RI: Recognition Index
TLR4: Toll-like receptor 4
WB: Western blot
GO: Gene ontology
DEGs: Differentially expressed genes
RNA-Seq: RNA sequencing
SPF: Specific Pathogen-Free
BBB: Blood-brain barrier
OTUs: Operational Taxonomic Units
PFA: Paraformaldehyde
HRP: Horseradish peroxidase
ANOVA: Analysis of variance
PCoA: Principal Coordinates Analysis
ANOSIM: Analysis of Similarities
LEfSe: Linear discriminant analysis Effect Size
SCFA: Short-chain fatty acid
