# Supplementary material for: Gut microbial dysbiosis exacerbates long-term cognitive impairments by promoting intestinal dysfunction and neuroinflammation following neonatal hypoxia-ischemia
Source: Gut Microbes. 2025 Feb 26;17(1):2471015. doi: 10.1080/19490976.2025.2471015 (PMC11866968; doi:10.1080/19490976.2025.2471015)
Supplement: Supplementary Information R3.docx [file KGMI_A_2471015_SM7223.docx]

**Supplementary Information for**

**“Gut microbial dysbiosis exacerbates long-term cognitive impairments by promoting intestinal dysfunction and neuroinflammation following neonatal hypoxia-ischemia”**

**Author:**

Andi Chen^a*^, Chengqian Teng^a*^, Jianjie Wei^a*^, Xuyang Wu^a^, Honghong Zhang^a^, Pinzhong Chen^a^, Dingliang Cai^a^, Haitao Qian^a^, Hui Zhu^b^, Xiaochun Zheng^a✉^, Xiaohui Chen^a✉^

**Affiliations:**

^a^Shengli Clinical Medical College of Fujian Medical University, Department of Anesthesiology, Fujian Provincial Hospital, Fuzhou University Affiliated Provincial Hospital, Fuzhou, China.

^b^Neonatal Intensive Care Unit of Fujian Provincial Hospital, Fuzhou, China

Andi Chen, Chengqian Teng, and Jianjie Wei have contributed equally to this work.

^✉^**Correspondence:** [zhengxiaochun@fjsl.com](mailto:zhengxiaochun@fjsl.com) (Xiaochun Zheng) and [cxh19870305@163.com](mailto:cxh19870305@163.com) (Xiaohui Chen).

**Table of Contents:**

(1). Supplementary Notes

(2). Supplementary Tables

(3). Supplementary Figures

**Supplementary Notes**

**Supplementary Note 1.**

Previous study[1] indicated that a 1 mg/kg oral dose of dexamethasone (DEX) is safe in mouse models of other neurological diseases and beneficial for gut microbiota, intestinal function, and cognitive function. Accordingly, we established a doubling gradient scheme including five doses (low to high): 0.25 mg/kg, 0.5 mg/kg, 1 mg/kg, 2 mg/kg, and 4 mg/kg. The administration regimen in neonatal hypoxic-ischemic brain damage (HIBD) rat model consisted of daily dosing from the day of hypoxic-ischemic (HI) insult through the third day. This study firstly focused on the impact of different-dose DEX on intestinal inflammation post-HI insult by evaluating the expression of the inflammatory mediator IL-17a in intestinal tissue using immunohistochemistry. The Known side effects of DEX in neonates and children include growth retardation and neurotoxicity[2, 3]. Thus, we recorded body weight changes and observed hair condition to assess neonatal rat development and employed the Cliff aversion test for neurological function evaluation. In the pilot study, the neonatal rats were divided into seven groups: sham group, HI group, and five oral DEX treatment groups with different doses, with n=3 per group. Following the completion of oral DEX administration, observations continued for 7 days. On the 7th day, the cliff aversion test was performed, after which intestinal tissues were collected for immunohistochemical staining. Results indicated that compared with HI group, 0.25 mg/kg and 0.5 mg/kg doses did not significantly affect IL-17a expression, while 1 mg/kg dose significantly inhibited IL-17a. Although higher doses of 2 mg/kg and 4 mg/kg showed stronger inhibition, no statistically significant differences existed between them (Figure S1a, b). Regarding side effects, HI group rats demonstrated poorer growth and neurobehavioral performance compared to the Sham group, indicated by reduced body weight in day 10 after HI insult, sparse hair growth, and significantly prolonged cliff avoidance response time. In comparison to the HI group, the 0.25 mg/kg and 0.5 mg/kg dose groups showed no differences in these parameters, the 1 mg/kg group showed improvement, while the 2 mg/kg group showed no differences in growth but mild neurobehavioral deterioration, and the 4 mg/kg group performed worse in all these aspects (Figure S1c-e). Integrating the report of previous study with our pilot study results, we considered that a 1 mg/kg oral dose of DEX, administered daily from the day of HI insult to the third day, was a safe and effective treatment for neonatal rat model of HIBD. Furthermore, our main findings (Figure 9, S3, 10) confirm that this dosage and method of oral DEX administration can improve gut microbiota dysbiosis, intestinal dysfunction, systemic inflammatory response, brain injury, and long-term cognitive impairments in neonatal HIBD rats.

**Supplementary Note 2.**

Previous studies[4, 5] have demonstrated that a dose of 1 mg/kg of TLR4-IN-C34 is both safe and effective in animal models of necrotizing enterocolitis and ulcerative colitis, significantly improving intestinal inflammation. Notably, the administration method and dosage of TLR4-IN-C34 in neonatal HIBD rats have not been previously documented. According to literature reports and our experience with exploring effective dose of oral DEX, we implemented three dosage levels through a tripling method (low to high): 0.3 mg/kg, 1 mg/kg, and 3 mg/kg. The dosing schedule remained once daily from the day of HI insult to the third day. We assessed the efficacy of different-dose TLR4-IN-C34 treatment by immunofluorescence detection of TLR4 expression in intestinal tissues and monitored body weight changes, hair condition, and used the Cliff Aversion Test to evaluate changes in neurological function. In the pilot experiments, neonatal rats were divided into five groups: sham group, HI group, and three oral TLR4-IN-C34 treatment groups with different doses, with n=3 per group. Following the completion of oral TLR4-IN-C34 administration, observations continued for 7 days. On the 7th day, the cliff aversion test was performed, after which intestinal tissues were collected for immunofluorescence staining. Results indicated that, compared to the HI group, the 0.3 mg/kg group showed no significant impact on TLR4 expression, but 1 mg/kg group had a significantly inhibited TLR4 expression and the 3 mg/kg group exhibited an even stronger inhibitory effect (Figure S2a, b). Regarding side effects, HI group rats demonstrated poorer growth and neurobehavioral performance compared to the Sham group, indicated by reduced body weight in day 10 after HI insults, sparse hair growth, and significantly prolonged cliff avoidance response time. In comparison to the HI group, the 0.3 mg/kg dose groups showed no differences in these parameters, both the 1 mg/kg and 3 mg/kg groups exhibited significant improvements, yet no statistical differences were observed between them (Figure S2c-e). Although our pilot results indicated a stronger inhibition of TLR4 expression in the intestinal tissues with a 3 mg/kg dose, no additional side effects were noted. As TLR4 is a key component of the immune system[6], there may be other unobserved side effects in our pilot study. Based on the results of previous studies and our pilot experimental results, we selected the same safe and effective lower dosage, specifically an oral dose of TLR4-IN-C34 1 mg/kg, for our study. Furthermore, our main findings (Figure 11) confirmed that this dosage and method of oral TLR4-IN-C34 administration can significantly inhibited TLR4 expression in the intestinal tissue of neonatal HIBD rat model, regulated intestinal function, improved systemic inflammation, synaptic damage, and long-term cognitive impairments.

**References for Supplementary Notes:**

1. Pan C, Zhang H, Zhang L, Chen L, Xu L, Xu N, et al. Surgery-induced gut microbial dysbiosis promotes cognitive impairment via regulation of intestinal function and the metabolite palmitic amide. Microbiome. 2023, 11(1):248.

2. Concepcion KR, Zhang L Corticosteroids and perinatal hypoxic-ischemic brain injury. Drug discovery today. 2018, 23(10):1718-1732.

3. Buchiboyina AK, Yip CSA, Kohan R, Nathan EA, Shrestha D, Davis J, et al. Effect of cumulative dexamethasone dose in preterm infants on neurodevelopmental and growth outcomes: a Western Australia experience. Archives of disease in childhood Fetal and neonatal edition. 2021, 106(1):69-75.

4. Chen Y, Li D, Sun L, Qi K, Shi L Pharmacological inhibition of toll-like receptor 4 with TLR4-IN-C34 modulates the intestinal flora homeostasis and the MyD88/NF-κB axis in ulcerative colitis. European journal of pharmacology. 2022, 934:175294.

5. Neal MD, Jia H, Eyer B, Good M, Guerriero CJ, Sodhi CP, et al. Discovery and validation of a new class of small molecule Toll-like receptor 4 (TLR4) inhibitors. PloS one. 2013, 8(6):e65779.

6. Liu Y, Yang M, Tang L, Wang F, Huang S, Liu S, et al. TLR4 regulates RORγt(+) regulatory T-cell responses and susceptibility to colon inflammation through interaction with Akkermansia muciniphila. Microbiome. 2022, 10(1):98.

**Supplementary Tables**

**Supplementary Table 1**. The number of animal samples used in pilot study.

|  | **Experimental objective** | Mortality | Total number |
| --- | --- | --- | --- |
| Pilot study | To determine the safe and effective doses and methods of DEX and TLR4-IN-C34 for oral administration in neonatal HIBD rats | 6 | 42 |

HIBD, hypoxic-ischemic brain damage; DEX, dexamethasone.

**Supplementary Table 2**. The number of animal samples used in different experimental groups at various experimental stages in main study.

| **Experimental objective** | **Grouping and the number of neonatal rats in each group** | | | | | | | **Mortality and total number of used neonatal rats in each experimental objective** | |
| --- | --- | --- | --- | --- | --- | --- | --- | --- | --- |
| 1. To determine the association between gut microbiota and cognitive impairments after HI insult. | Sham | | | | HI | | | Mortality | Total number |
|  | 8 | | | | 8 | | | 2 | 18 |
| 2. To explore the potential microbiota-gut-brain axis mechanisms underlying cognitive impairments after HI insult. | Sham | | | | HI | | | Mortality | Total number |
|  | 5 | | | | 5 | | | 2 | 12 |
| 3. FMT was performed to validate the causal relationship between gut microbiota and cognitive impairments after HI insult. | Donor rats | | | | Recipient rats | | | Mortality | Total number |
|  | 52 | | | | 52 | | | 15 | 119 |
| 4. To exprole the effect of oral DEX treatment on neonatal HIBD rats. | Sham | | | HI | | HI+DEX | | Mortality | Total number |
|  | 13 | | | 13 | | 13 | | 7 | 46 |
| 5. FMT was performed to validate whether the gut microbiota was a key mediator for the neuroprotective effect of oral DEX treatment | Donor rats | | | | Recipient rats | | | Mortality | Total number |
|  | 26 | | | | 26 | | | 11 | 63 |
| 6. To further determine the role of LPS/TLR4 pathway in neonatal HIBD rats. | Sham | HI | HI+TLR4-IN-C34 | | HI+shamFMT and donor rats | | HI+DEX | Mortality | Total number |
|  | 19 | 19 | 19 | | 12 | | 6 | 10 | 85 |
| **Total number of used neonatal rats in this study** | | | | | | | | | 343 |
| **Total number of used dams in this study** | | | | | | | | | 33 |

HIBD, hypoxic-ischemic brain damage; FMT, fecal microbiota transplantation; DEX, dexamethasone; HI, hypoxic-ischemic;

**Supplementary Figures**


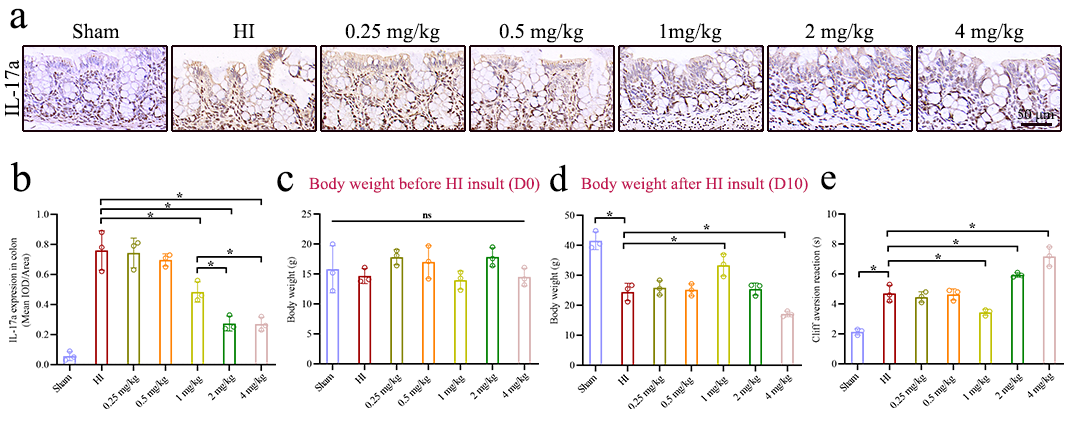


**Figure S1.** Effects of different-dose oral DEX on neonatal HIBD rats. a, b The expression levels of the IL-17a in the colons with the IHC staining and the mean IOD/area of this cytokines. c, d Body weight measurements were taken before HI insult and again on the 10th day after HI insult, following the completion of both the HI insult and oral DEX administration, and the data were then recorded. e On the 10th day after HI insult, the Cliff Aversion Test was conducted, and the reaction time was recorded. HI, hypoxic-ischemic; HIBD, hypoxic-ischemic brain damage; DEX, dexamethasone; IHC staining, immunohistochemical staining; IOD, integrated optical density; n = 3, per group; **P* < 0.05.


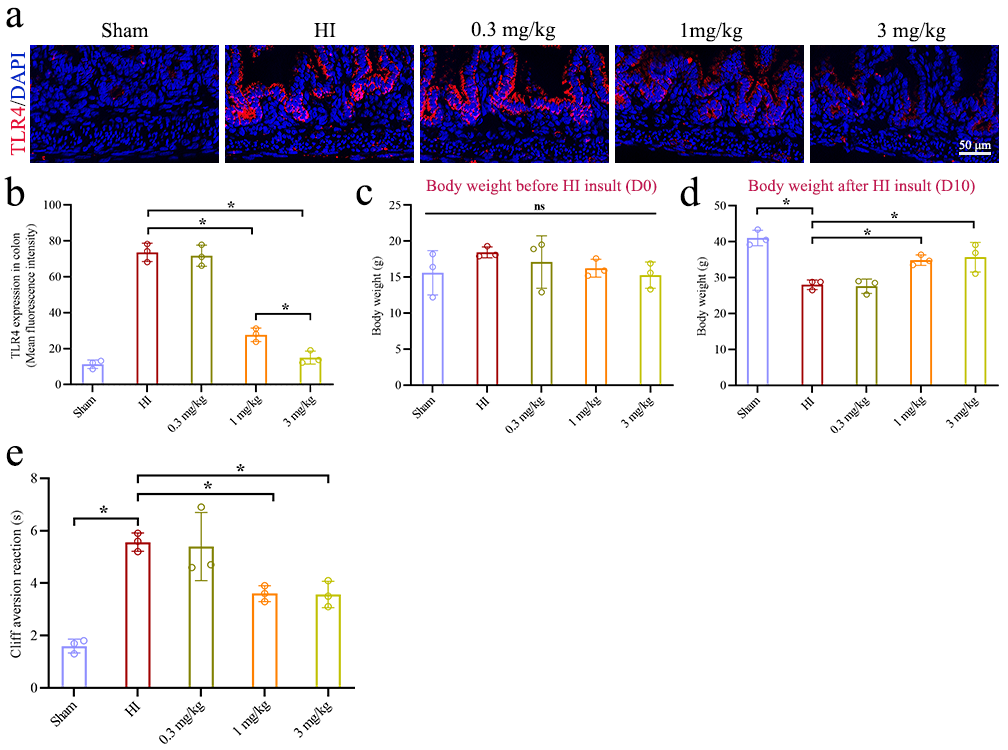


**Figure S2.** Effects of different-dose oral TLR4-IN-C34 on neonatal HIBD rats. a, b The expression levels of the TLR4 in the colons with the IF staining and the mean fluorescence intensity. c, d Body weight measurements were taken before HI insult and again on the 10th day after HI insult, following the completion of both the HI insult and oral TLR4-IN-C34 administration, and the data were then recorded. e On the 10th day after HI insult, the Cliff Aversion Test was conducted, and the reaction time was recorded. HI, hypoxic-ischemic; HIBD, hypoxic-ischemic brain damage; IF, immunofluorescence; n = 3, per group; **P* < 0.05.


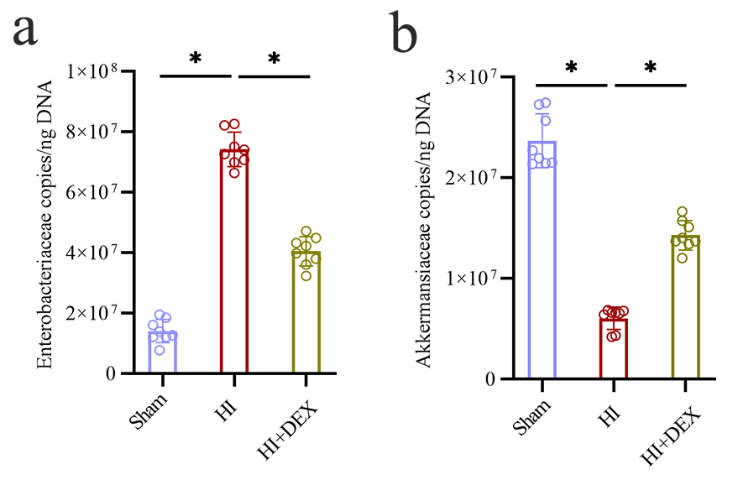


**Figure S3.** The effects of oral DEX treatment on the gut microbiota in the neonatal HIBD rats. a, b Quantification of *f_Enterobacteriaceae* and *f_Akkermansiaceae* in the fecal samples. HI, hypoxic-ischemic; HIBD, hypoxic-ischemic brain damage; DEX, dexamethasone; n = 8, per group; **P* < 0.05.


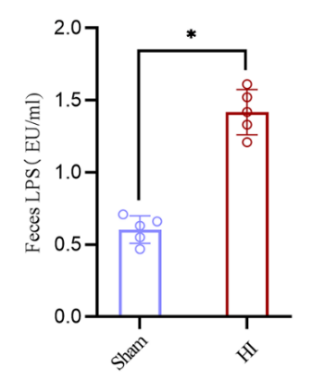


**Figure S4.** HI insult induced a significant increase of feces LPS levels in the neonatal HIBD rats. HIBD, hypoxic-ischemic brain damage; HI, hypoxic-ischemic; LPS, lipopolysaccharide. n = 5, per group; **P* < 0.05.


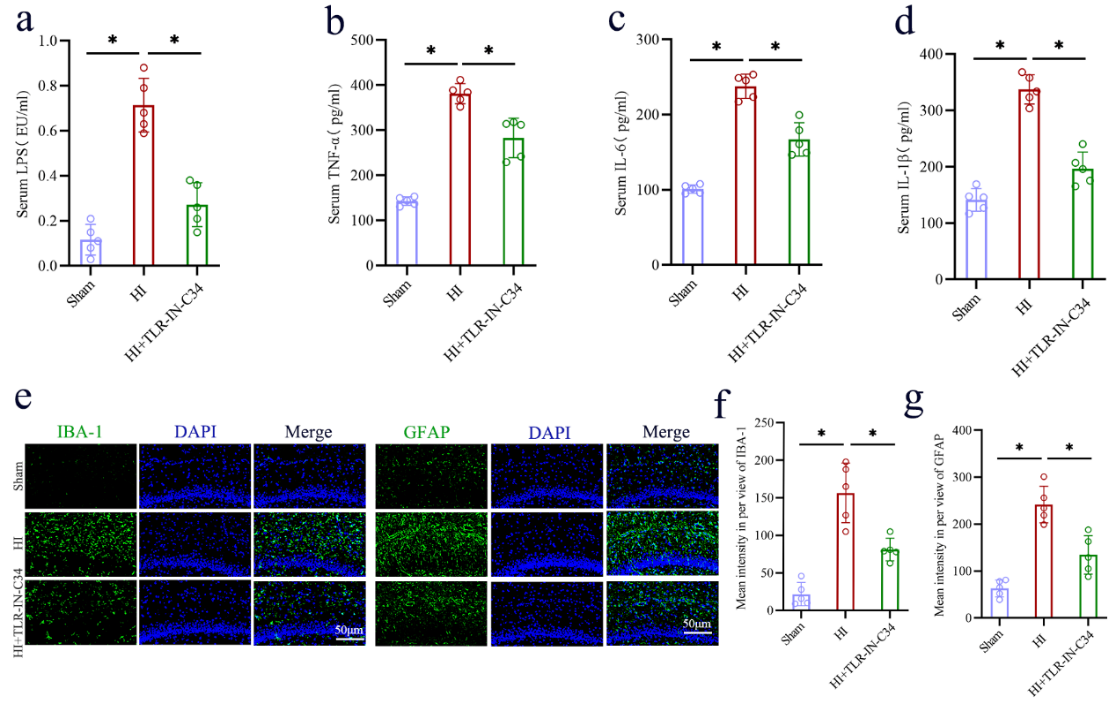


**Figure S5.** Oral TLR4-IN-C34 treatment reduced systemic inflammation in the neonatal HIBD rats. **a**-**d** The serum concentrations of LPS, TNF-α, IL-6, and IL-1β. **e** The expression levels of the microglial marker IBA-1 (green) and astrocyte marker GFAP (green) with the IF staining in the CA1 region of hippocampus. **f, g** The mean intensity in per view of IBA-1 and GFAP in the CA1 region of hippocampus. TLR4, toll-like receptor 4; HIBD, hypoxic-ischemic brain damage; HI, hypoxic-ischemic; n = 5, per group; **P* < 0.05.


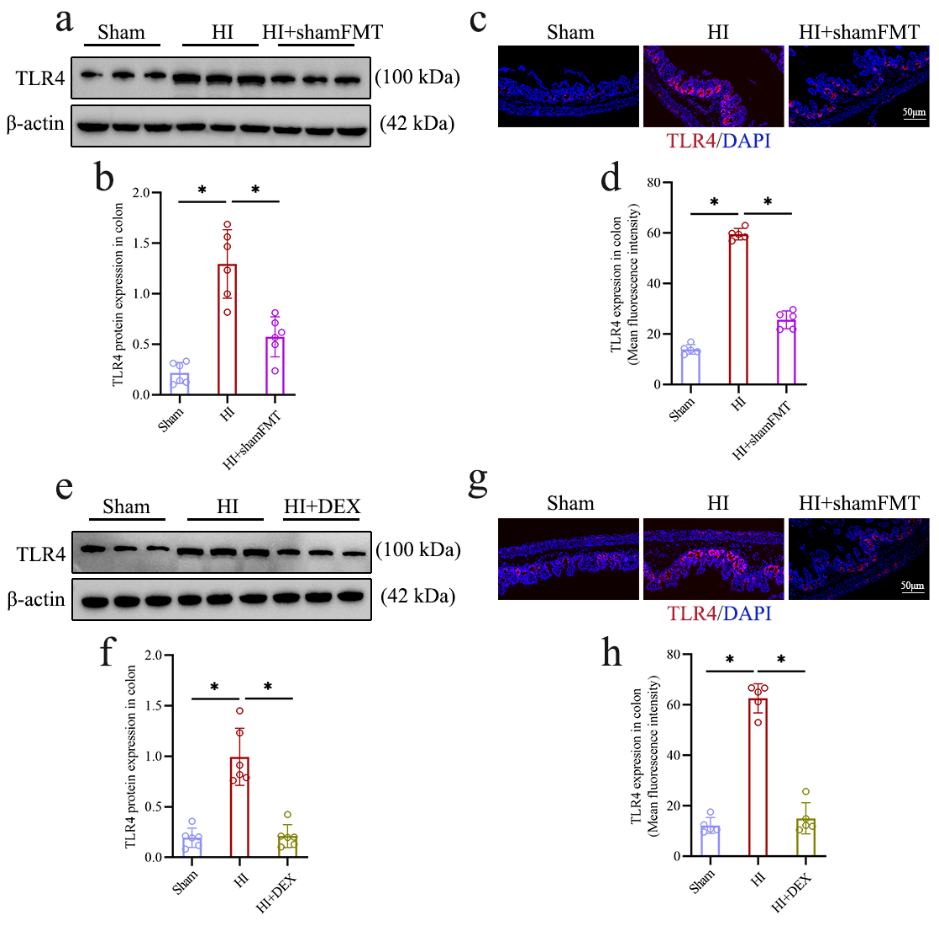


**Figure S6.** Both FMT treatment from cognitively normal rats and oral DEX treatment inhibited the expression of intestinal TLR4 in the neonatal HIBD rats. **a**-**h** The expression levels of TLR4 of colon with the WB and IF analyses. HIBD, hypoxic-ischemic brain damage; HI, hypoxic-ischemic; DEX, dexamethasone; FMT, fecal microbiota transplantation; TLR4, toll-like receptor 4; WB, western blot; IF, immunofluorescence. n = 5 or 6, per group; **P* < 0.05.


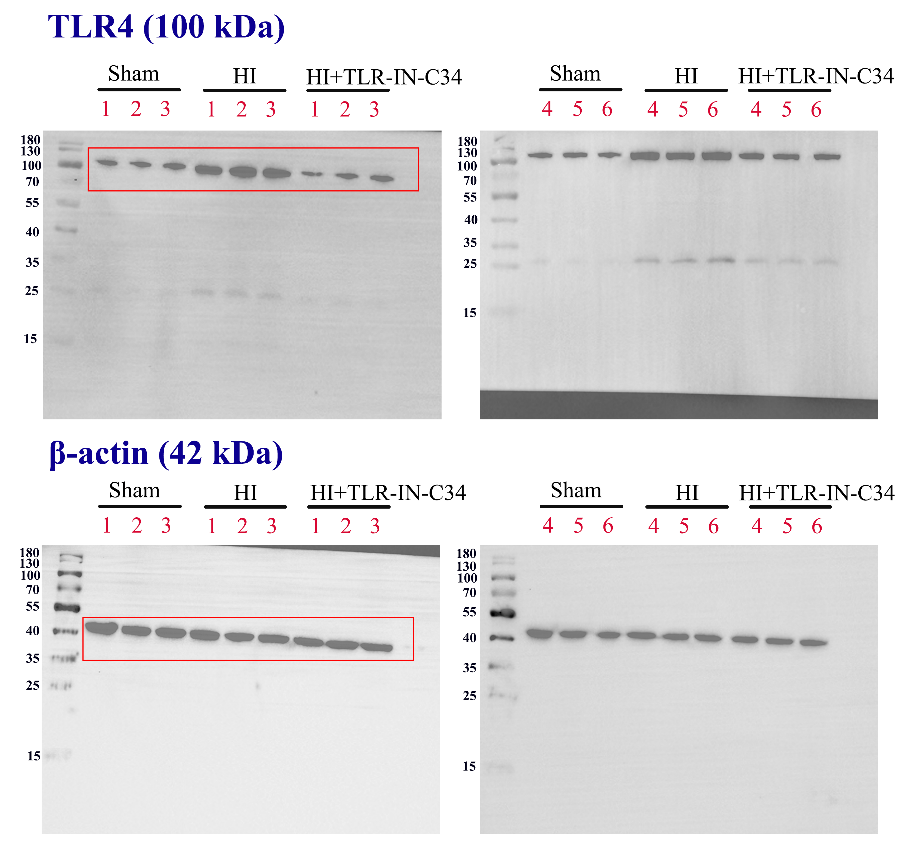


**Figure S7.** Full and unedited western blot images corresponding to Figure 11e, the red squares refer to the blots cited in the main article. HI, hypoxic-ischemic; n = 6, per group.


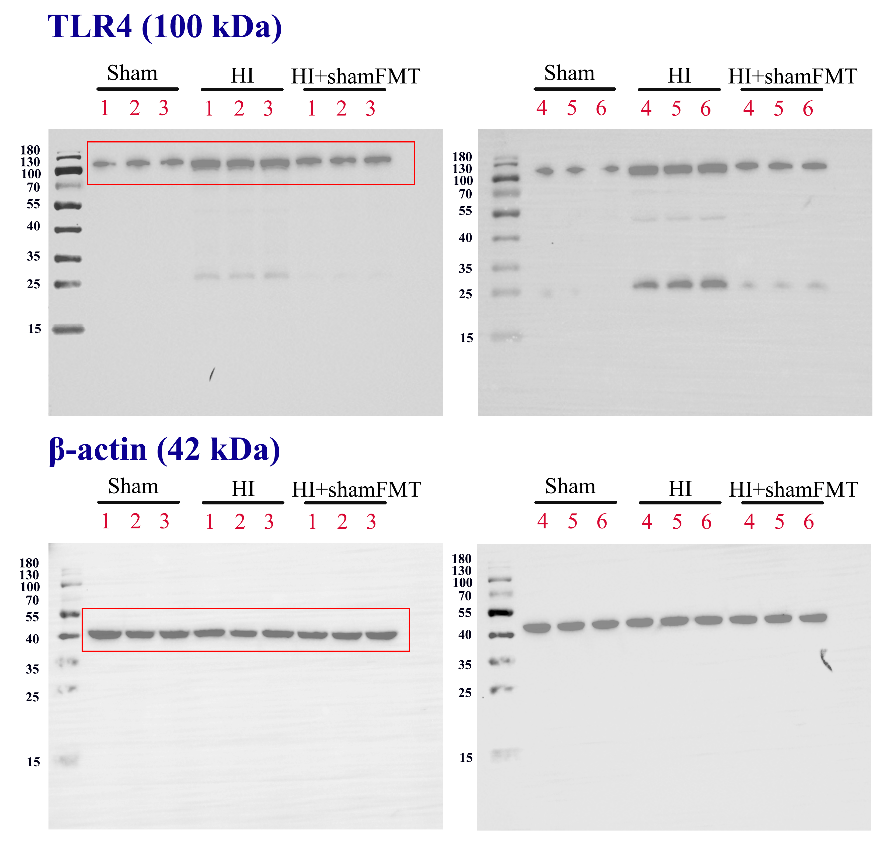


**Figure S8.** Full and unedited western blot images corresponding to Figure S4a, the red squares refer to the blots cited in the main article. HI, hypoxic-ischemic; FMT, fecal microbiota transplantation; n = 6, per group.


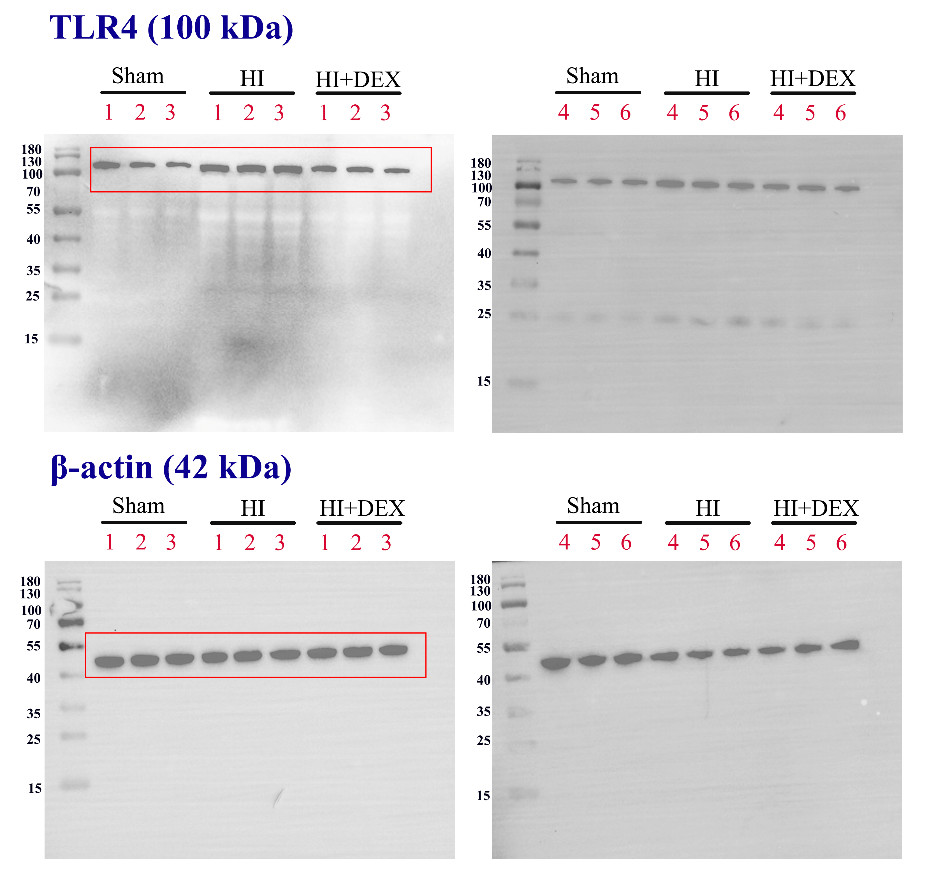


**Figure S9.** Full and unedited western blot images corresponding to Figure S4e, the red squares refer to the blots cited in the main article. HI, hypoxic-ischemic; DEX, dexamethasone; n = 6, per group.
